# Live-Cell Microscopy Reveals Small Molecule Inhibitor Effects on MAPK Pathway Dynamics

**DOI:** 10.1371/journal.pone.0022607

**Published:** 2011-08-04

**Authors:** Daniel J. Anderson, Jenni K. Durieux, Kyung Song, Ryan Alvarado, Peter K. Jackson, Georgia Hatzivassiliou, Mary J. C. Ludlam

**Affiliations:** 1 Department of Cell Regulation, Genentech, Inc., South San Francisco, California, United States of America; 2 Department of Cancer Signaling, Genentech, Inc., South San Francisco, California, United States of America; Ohio State University, United States of America

## Abstract

Oncogenic mutations in the mitogen activated protein kinase (MAPK) pathway are prevalent in human tumors, making this pathway a target of drug development efforts. Recently, ATP-competitive Raf inhibitors were shown to cause MAPK pathway activation via Raf kinase priming in wild-type BRaf cells and tumors, highlighting the need for a thorough understanding of signaling in the context of small molecule kinase inhibitors. Here, we present critical improvements in cell-line engineering and image analysis coupled with automated image acquisition that allow for the simultaneous identification of cellular localization of multiple MAPK pathway components (KRas, CRaf, Mek1 and Erk2). We use these assays in a systematic study of the effect of small molecule inhibitors across the MAPK cascade either as single agents or in combination. Both Raf inhibitor priming as well as the release from negative feedback induced by Mek and Erk inhibitors cause translocation of CRaf to the plasma membrane via mechanisms that are additive in pathway activation. Analysis of Erk activation and sub-cellular localization upon inhibitor treatments reveals differential inhibition and activation with the Raf inhibitors AZD628 and GDC0879 respectively. Since both single agent and combination studies of Raf and Mek inhibitors are currently in the clinic, our assays provide valuable insight into their effects on MAPK signaling in live cells.

## Introduction

The mitogen activated protein kinase (MAPK) pathway containing Raf, Mek and Erk is a central downstream axis of Ras signaling involved in Ras-driven transformation [1], [2]. Ras and Raf harbor activating mutations in 30% and 8% of human tumors, respectively [3], [4], [5], making these oncoproteins critical targets for oncology drug development.

Inhibitors of both Mek and Raf are currently in clinical trials. Although Mek inhibitors have shown little benefit in the clinic, likely due to a narrower therapeutic index [6], the BRafV600E selective inhibitor PLX4032 has demonstrated strong efficacy in treating metastatic melanoma [7]. Interestingly, incidences of squamous cell carcinoma and keratocanthoma have been reported in clinical trials of two selective Raf inhibitors [8], suggesting a potential growth-promoting effect of these agents in BRaf wild type (WT) tissues. Three recent studies have investigated such ATP-mimetic Raf inhibitors in BRaf-WT cells, showing that these inhibitors have the ability to activate MAPK signaling in cells with WT BRaf [9], [10], [11]. This activation is attributed to inhibitor-induced priming of the Raf kinase as indicated by Raf dimerization, targeting of Raf to plasma membrane (PM)-localized Ras and subsequent downstream MAPK pathway activation. Although there is significant promise in targeting the MAPK pathway as a therapeutic strategy, the effects of small molecule kinase inhibitors on normal and tumor cells must be well understood to ensure success in the clinic.

Inactive Raf is located in the cytosol, but upon Ras activation, Raf is recruited to the PM by Ras-GTP resulting in Raf activation. Raf membrane translocation can act as a reliable reporter for Ras activation. Classically, the Ras binding domain of Raf (RBD), which binds selectively to GTP-bound Ras, has been used to biochemically measure the extent of Ras activation via pull-down experiments [12]. More recently, fluorescent protein fusions of RBD or full-length Raf have been used to visualize Ras activation through PM translocation of these reporter constructs [13], [14], [15]. Although imaging approaches provide a real-time readout of Ras activity, only limited manual quantification and low-throughput acquisition strategies to visualize Ras activation have been implemented, making these approaches limited in scope. Other microscopy-based approaches have been developed using fluorescent resonance energy transfer (FRET) between fluorescently-labeled Ras and GTP or RBD, or conformational changes in a dual-fluorescently labeled Ras-RBD fusion [16], [17], [18]. Although these assays have the advantage of direct biophysical detection of Ras binding, they have yet to be implemented in large-scale studies.

In this study we have extended the capabilities of a Ras-driven Raf redistribution assay by fluorescently labeling both Ras and Raf in an inducible, bicistronic system, a critical step in automating the detection of Ras activation and high-throughput analysis. We developed novel image analysis protocols to facilitate the increased scale of these assays and scope of our study. We first characterized a fragment of Raf containing the Ras binding domain and a cysteine-rich domain, RBDCRD that shows high sensitivity for activated Ras and can detect changes in endogenous Ras activity. We further expanded the redistribution assay by adding fluorescently labeled Mek and Erk to visualize the complete canonical MAPK pathway, with Erk nuclear translocation serving as a readout of downstream pathway activity. Finally, we applied these assays to a systematic study of the effect of hundreds of dosing conditions of Raf, Mek and Erk inhibitors alone and in combination in order to better understand how these small molecule inhibitors affect pathway activity.

## Results

### Building an image analysis platform for measuring Ras activation

To better understand the mechanistic outcome of therapeutically targeting components of the MAP-kinase pathway, we set out to develop a quantitative method for measuring Ras activation by monitoring the interaction between Ras and Raf. KRas, one of three Ras isoforms, was used because it is commonly mutated in cancer and is targeted exclusively to the PM [19], simplifying subsequent analyses. eCFP-KRas and Venus-CRaf were co-transfected into HEK 293T cells and imaged via confocal microscopy to collect cross-sectional images of cells. We achieved good spatial resolution covering the equatorial PM and the adjacent cytoplasm, a critical approach for subsequent automated analysis (Figure S1a). As expected, both RBD (aa51-131) and full-length CRaf showed little targeting to the PM when cotransfected with WT KRas, but higher membrane localization when these constructs were cotransfected with oncogenic KRasG12D (Figure S1b). These data confirmed that either RBD or full-length CRaf can act as detectors of over-expressed activated KRas in live cells.

**Figure 1:**
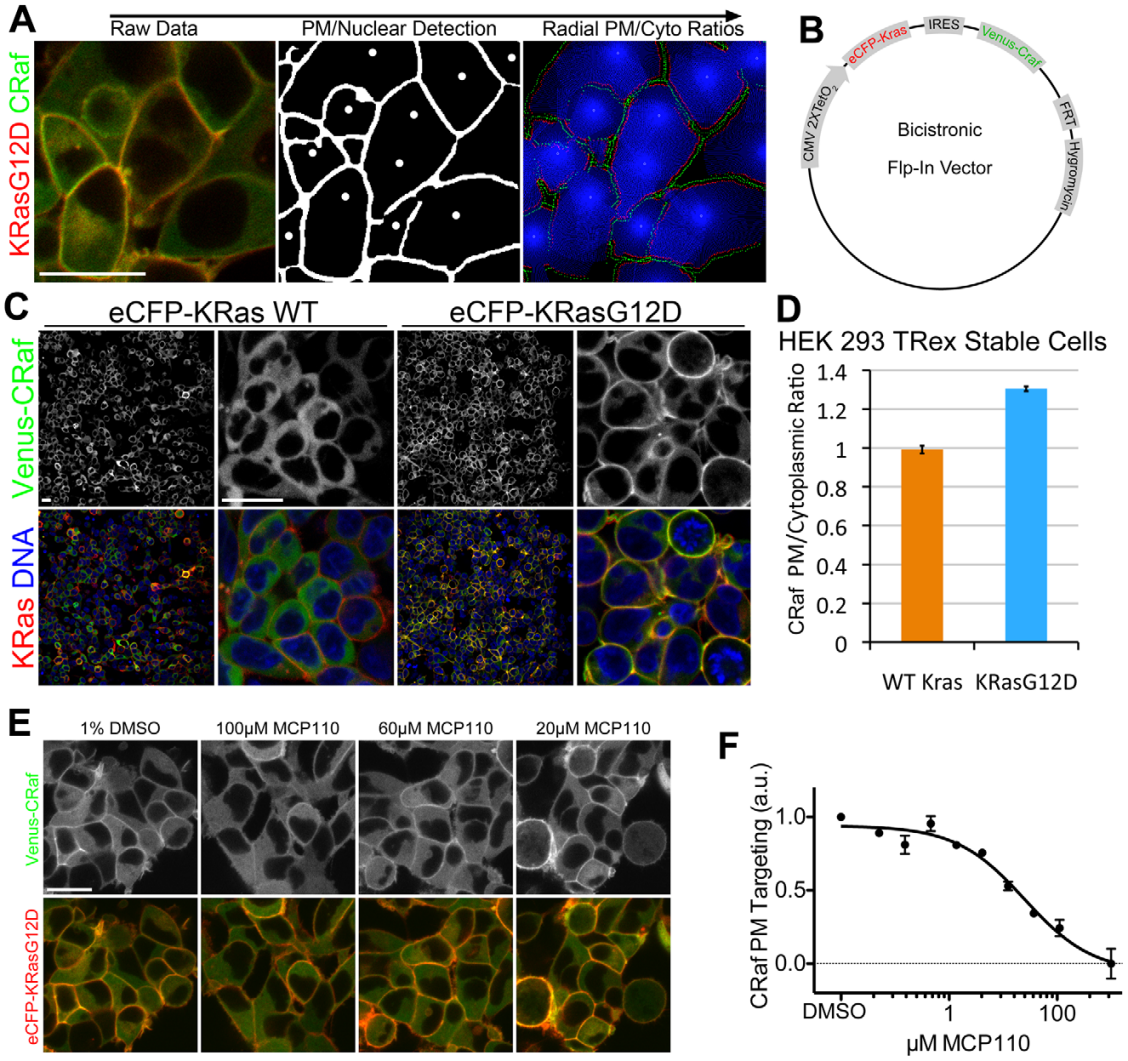
Automated Ras-mediated Raf redistrbution analysis. (**A**) Graphical representation of raw data from Raf-redistribution assay analysis (left, mCherry-KRas in red, Venus-CRaf in green) nuclear and PM masking (center) and radial intensity ratio scanning (right) show image analysis approach. Scale bar is 20 µm and applies to all panels. (**B**) Schematic of bicistronic vector used to generate stable cell lines. (**C**) Images of bicsitronic Flp-In stable cell lines expressing Venus-CRaf with eCFP-KRasWT and eCFP-KRasG12D. Cells were stained with Hoechst to demonstrate the percentage of cells that express the fluorescent-fusion proteins (>99% for both cell lines). Scale bars are 20 µm and apply to corresponding magnifications in all panels. (**D**) CRaf PM/cytoplasmic ratio measurement of KRasWT and KRasG12D stable cell line, p<0.001. (**E**) eCFP-KRasG12D and Venus-CRaf expressing cells were treated with a serial dilution of MCP110 between 100 µM and 24 nM for 2.5 hrs, example images shown. Scale bar is 20 µm and applies to all panels. (**F**) CRaf PM/cytoplasmic intensity ratios were measured for MCP110 serial dilution in (E).

To accurately measure the targeting of signaling components that translocate to the PM upon activation, we developed an image analysis program that uses object-based filtering to selectively measure PM Ras activation. To accomplish this, we identified the centroids of cells (see Methods) and masked the PM using eCFP-KRas or PMem localization (Fig. 1a). Ratios of PM/cytoplasmic localization were measured by scanning outward from the nuclear centroid until reaching the PM, where a local pixel intensity ratio was recorded, and this procedure was repeated radially around each cell (Figure 1a, right panel). Our approach allowed for the detection of very subtle changes in PM localization by measuring local differences in intensity, thus decreasing the effect of large-scale intensity variations and making the approach amenable to high-content imaging studies. When cells transiently co-transfected with eCFP-KRas and Venus-CRaf were subjected to this pixel ratio measurement approach, oncogenic KRasG12D caused an increase in both RBD and FL-CRaf targeting to the PM (Figure S1b). We further tested the sensitivity of our membrane targeting analysis program using HEK 293 cells that stably expressed Akt1-Venus and a fluorescent PM reporter (eCFP-PMem) (Figure S2). Although Akt1 visually showed only very subtle PM targeting in a serum-dependent manner [20], analysis using the pixel ratio program measured a significant increase in PM targeting from 1.08 to 1.11 (p = 0.019). Taken together, our image analysis program allows for automated detection of PM localization of fluorescently labeled proteins, facilitating large-scale quantitative imaging-based studies.

Since the stoichiometric balance of Ras and Raf expression was important for robust targeting of Raf to the PM (data not shown), we designed an expression system that would allow for stable, physiologically relevant and balanced expression of Ras and Raf. This system includes an internal ribosomal entry sequence (IRES) element for bicistronic expression of two open reading frames, a Tet-On CMV promoter for inducible expression and an FRT recombination site, allowing single integration events to generate stable cells (Fig. 1b). Inducible expression was required to generate a stable HEK 293 cell line expressing KRasG12D, since prolonged constitutive expression was cytotoxic (data not shown). Inducible cell lines were generated for both WT eCFP-KRas and eCFP-KRasG12D with Venus-CRaf (Fig. 1c). When the PM/cytoplasmic ratio of these cell lines was measured, the standard deviation in our measurement of PM targeting was much lower than that of transiently transfected cells, with estimated Z-factors of 0.69 and 0.09 respectively (Fig. 1d and Figure S1). In summary, our inducible bicistronic FRT recombination vector system allows for regulated, balanced and homogeneous expression of multiple components of a signaling pathway, making it ideal for interrogating their behavior in response to perturbation.

To validate the eCFP-KRasG12D/Venus-CRaf bicistronic stable cell line and our automated analysis methods for the investigation of PM targeting upon perturbations by small molecule inhibitors, cells were dosed with a 10-point dilution curve of MCP110, which was recently shown to disrupt Raf targeting to Ras at the PM [21]. Using our system we were able to calculate that MCP110 blocked PM recruitment of CRaf at an EC_50_ of 5.1 µM with an R^2^ value of 0.963 (Fig. 1e and f), consistent with previous results.

### RBDCRD senses endogenous Ras GTP loading

**Figure 2:**
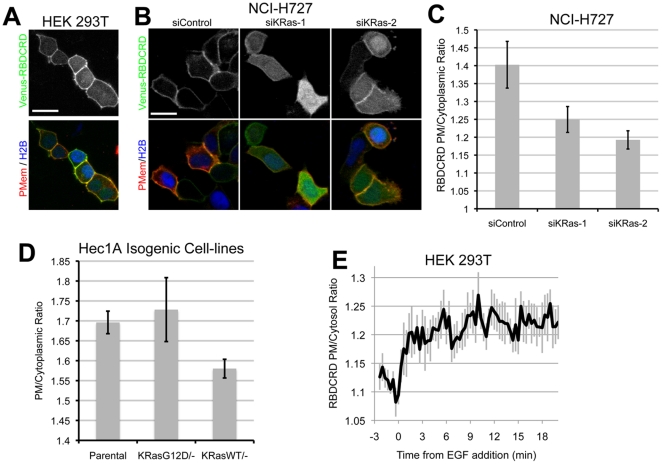
CRaf RBCRD detects endogenous RasGTP. (**A**) HEK 293T cells were transfected with Venus-RBDCRD, eCFP-CAAX PM reporter (PMem), and mCherry-H2B to label nuclei. Scale bar is 20 µm and applies to all panels. (**B**) KRas protein levels were reduced in NCI-H727 cells using 2 different siRNA oligos against KRas or control oligo and then transfected with Venus-RBDCRD. Scale bar is 20 µm and applies to all panels. (**C**) Quantification of RBDCRD PM targeting in NCI-H727 with KRas siRNA. (**D**) Hec1A isogenic cell ines (Parental, Ras^WT/-^ and Ras^G12D/-^) were transfected with Venus-RBDCRD and imaged live. RBDCRD PM targeting was then measured for each cell line. (**E**) Serum starved HEK 293T cells were stimulated with 100 ng/ml EGF, and imaged every 15 sec, PM targeting of RBDCRD was then measured.

When analyzing fragments of CRaf, we found strong PM targeting of a fragment containing the RBD and adjacent cysteine rich domain (RBDCRD, aa51-186) with both WT KRas and KRasG12D; however, targeting was impaired upon transfection with the dominant negative mutant KRasS17N (Figure S1b and 4c). In fact, when RBDCRD was transfected with only a PM reporter (CAAX motif), partial membrane targeting was detected with only endogenous WT Ras present (Fig. 2a). It has been suggested that this fragment of CRaf shows increased affinity for RasGTP due to the absence of the regulatory domain that is present in full-length CRaf [22]. Recent cellular data suggest that RBDCRD may be a sensor for endogenous Ras-GTP [13]; however, subsequent biophysical data have challenged this conclusion [23]. Given the potential utility of a live cell sensor for activated endogenous Ras, we set out to confirm the ability of RBDCRD to discriminate endogenous RasGTP from RasGDP. To determine whether RBDCRD targeting to the PM was Ras-dependent, NCI-H727 cells, which have amplified expression of KRasG12D [24], were transfected with Venus-RBDCRD. When KRas protein levels were reduced by siRNA (data not shown), RBDCRD PM targeting decreased, causing a notable increase in cytoplasmic intensity and suggesting the KRas-dependent targeting of RBDCRD (Fig. 2b,c). To test whether RBDCRD bound preferentially to RasG12D, isogenic Hec1A cells in which either the WT or G12D allele of KRas was deleted [25] were transfected with Venus-RBDCRD. PM targeting of RBDCRD was higher in the Hec1A^KRasG12D/−^ cell line when compared to the Hec1A^KRasWT/−^ cells, suggesting that RBDCRD does bind preferentially to activated KRas expressed at endogenous levels (Fig. 2d). We next generated a 293 TRex™ cell line expressing Venus-RBDCRD and mCherry-PMem (for PM masking in the absence eCFP-KRas expression). Due to lower steady state expression compared to transiently transfected cells, RBDCRD almost entirely targeted to the PM in these cells (Figure S4a and b). To demonstrate that membrane targeting was dependent on RasGTP levels, RBDCRD-expressing cells were transfected with eCFP-KRasS17N, a dominant negative KRas mutant that depletes endogenous RasGTP by sequestering Ras GEFs [26]. KRasS17N expression caused a decrease in RBDCRD targeting to the PM, which was further decreased by serum starvation (Figure S4b,c). We next stimulated serum starved 293T cells transfected with Venus-RBDCRD with epidermal growth factor (EGF), and monitored RBDCRD PM translocation using time-lapse imaging (Fig. 2e and Figure S4d). It has been suggested that RBDCRD remains locked on the membrane once recruited by Ras [23]. To test this in our system we dosed RBDCRD stable transfectants with MCP110 (Figure S5a and b). Displacement from the PM was seen at similar doses as for FL-Craf, suggesting that RBDCRD interaction with Ras at the PM can be disrupted. Together, these data confirm that RBDCRD does indeed act as a sensitive sensor of Ras GTP biding, allowing the monitoring of endogenous increases in Ras activity. The data also demonstrate the improved sensitivity of our imaging platform over existing imaging assays for monitoring physiologically relevant changes in RasGTP levels.

### Raf priming and MAPK feedback

**Figure 3:**
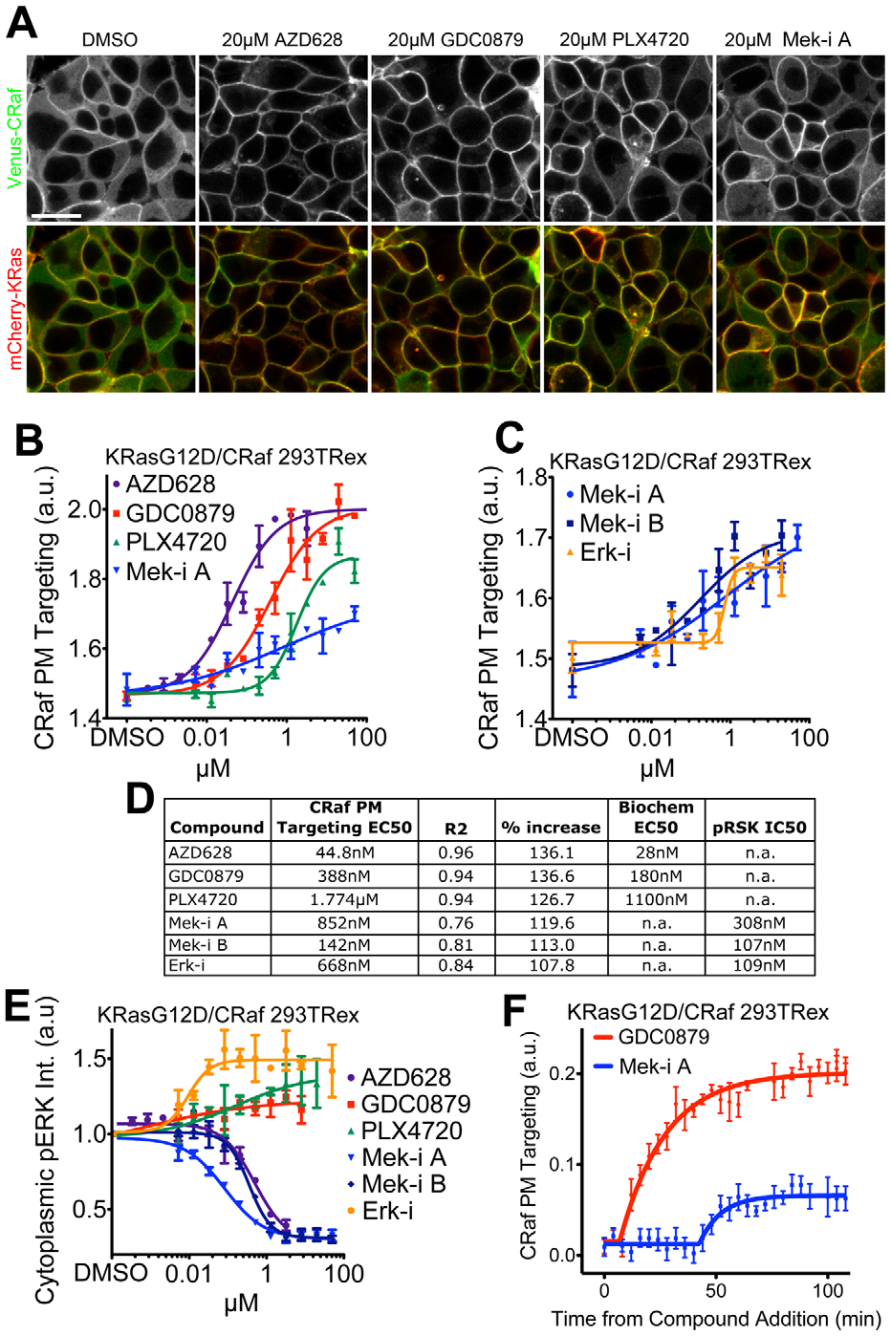
Raf priming and MAPK negative feedback inhibition act to recruit CRaf to the PM. (**A**) The Raf inhibitors AZD628, GDC0879 and PLX4720 along with Mek-i A were dosed in eCFP-KRasG12D and Venus-CRaf expressing HEK 293 cells, example images shown. Scale bar is 20 µm and applies to all panels. (**B**) PM/cytoplasmic CRaf intensity for images such as those in (A) was measured 4 hours after compound addition. (**C**) Mek-i A was compared to Mek-i B and Erk-i in CRaf redistribution assay. (**D**) Chart shows EC_50_ values for both the CRaf PM targeting assay and biochemical kinase activity. (**E**) Cytoplasmic pERK was measured in cells dosed wih Raf, Mek and Erk inhibitors by immuno-fluorescence. (**F**) Kinetics of Raf inhibitor priming and feedback were compared by conducting time lapse analysis of cells treated with GDC0879 and Mek-i A, frames acquired every 2 min.

We have previously used a qualitative, transient transfection Raf redistribution assay to characterize the priming effect of the ATP-mimetic Raf inhibitors GDC0879, PLX4720 and AZD628 on Raf PM targeting [9]. To better compare the potencies of these Raf inhibitors on PM targeting of Craf, we utilized the improved quantitative assay described above to calculate EC_50_s for each compound (Fig. 3b). For these studies we used our KRasG12D/CRaf cell line that showed an intermediate basal level of CRaf targeting to the PM that, in turn, facilitates monitoring of Raf recruitment to and displacement from the PM. While all Raf inhibitors tested induced CRaf PM targeting, in the case of GDC0879 and AZD628 targeting of CRaf to the PM appeared to be nearly complete (Fig. 3a). The relative potencies of Raf inhibitors on CRaf membrane targeting correlated well with their estimated biochemical potencies against CRaf at physiological ATP concentrations (Fig. 3d) [9]. Subtle, but significant, increases in PM targeting were also measured when cells were treated with the Mek inhibitor PD0325901 (Mek-i A) (Fig. 3b). To determine whether this effect was similar for other MAPK pathway inhibitors, we tested an additional Mek inhibitor (Mek-i B) [27] and an Erk inhibitor (Erk-i) [28] (Fig. 3c). Both Mek-i A and Mek-i B as well as the Erk-i caused a dose-dependent increase in CRaf PM targeting. We hypothesized that the recruitment of CRaf to the PM was due to releasing negative feedback that is triggered by active Erk, acting directly on CRaf as well as RasGTP levels [29], [30]. Indeed, both Mek and Erk inhibitors were effective in blocking downstream MAPK signaling in our engineered cell line at doses that induced CRaf PM translocation, as shown by immuno-fluorescence for phosphorylated ERK (Fig. 3e). As previously reported[9], the Raf inhibitors GDC0879 and PLX4720 caused an increase in pERK (Fig. 3e). Although AZD-628 induced CRaf PM targeting, CRaf activity and therefore pERK levels were decreased, which is consistent with our previous studies (Fig. 3e) [9]. As expected, while both Mek and Erk inhibitors effectively decreased phosphorylation of the ERK targets RSK (Thr359/Ser363) and CRaf (ser289, Ser290, Ser304), Raf inhibitors had no such effect (Figure S6 and data not shown). This confirms that our system is sensitive enough to allow monitoring of both Raf inhibitor priming and MEK/ERK inhibitor negative feedback release.

Next, we hypothesized that there might be kinetic differences in CRaf PM targeting caused by Raf priming versus negative feedback release. To test this, RasG12D/CRaf cells were imaged every 3 minutes after dosing with the Raf inhibitor GDC0879 or Mek-i A (Fig. 3f). PM targeting of CRaf occurred immediately after GDC0879 addition, whereas Mek-i A-induced PM targeting occurred after 36 minutes, suggesting that a switch-like accumulation of feedback release is required to induce CRaf PM targeting. Such switch-like responses are often dependent on relative concentrations of signaling components [31]. Accordingly, this observed delay in CRaf targeting resulting from feedback inhibition is reduced by increased expression level of Mek1 and Erk2 in a KRasG12D/CRaf context (Figure S7). In conclusion, although Raf priming and Mek/Erk feedback release both lead to Raf targeting to the PM, mechanistic differences between the two processes can be elucidated by localization-based studies and further refined by incorporating a temporal analysis into a high-content quantitative end-point analysis.

### Additive effect of priming and feedback inhibition

**Figure 4:**
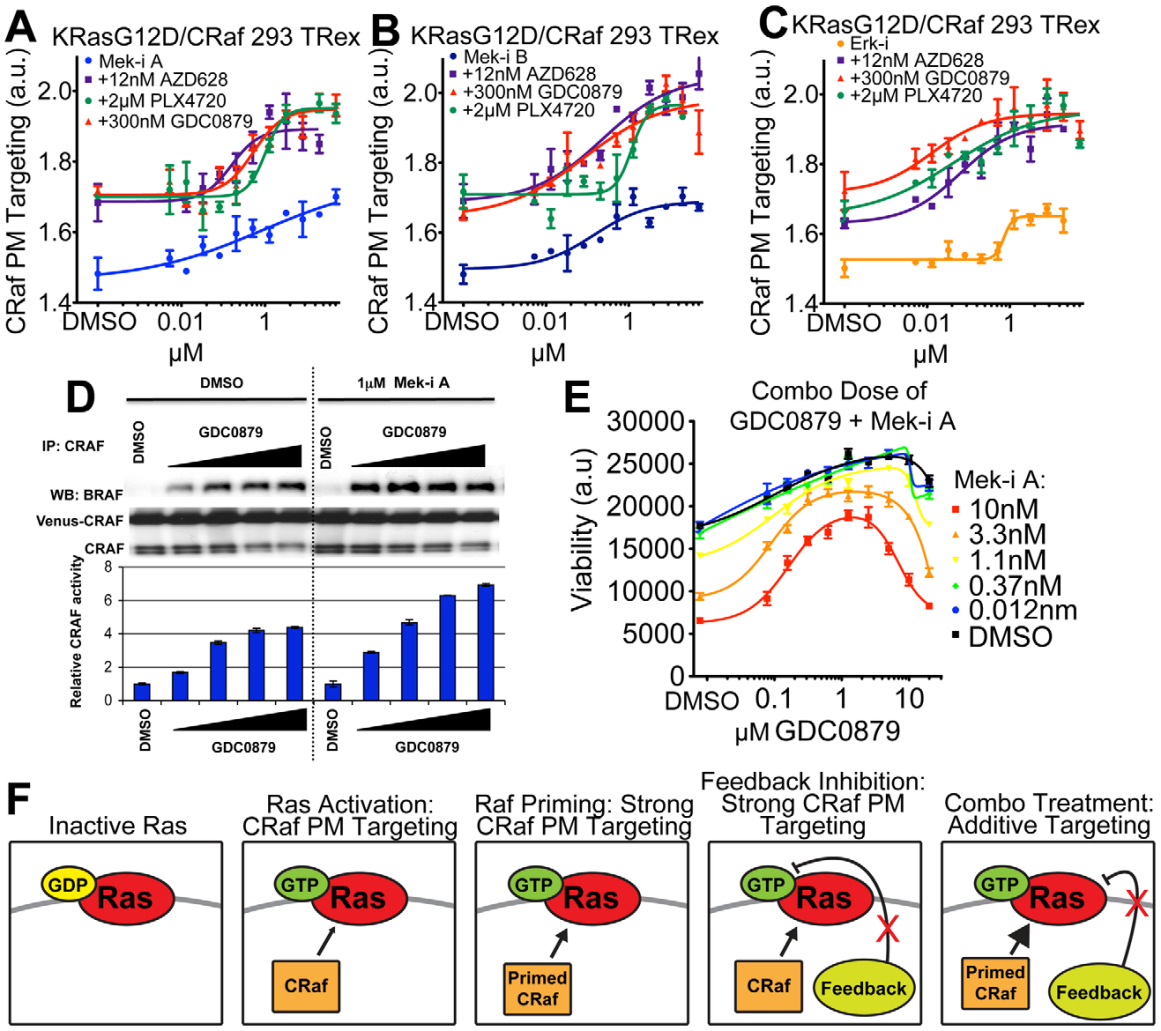
Combination of Raf priming with Mek/Erk inhibition has an additive effect on MAPK signaling. (**A**) KRasG12D/CRaf HEK 293 cells were dosed with varying concentration of Mek-i A and constant dose of 12 nM AZD628, 2 µM PLX4720 or 300 nM GDC0879, as indicated, and CRaf PM targeting was measured after 4 hrs. (**B**) CRaf PM targeting was measured for the combination of Mek-i B with 12 nM AZD628, 2 µM PLX4720 or 300 nM GDC0879. (**C**) CRaf PM targeting was measured for the combination of Erk-i with12 nM AZD628, 2 µM PLX4720 or 300 nM GDC0879. (**D**) Top: Immunoblot of Braf:Craf heterodimers in cells treated with GDC-0879 (0.03, 0.1, 0.3, 1 µM) for 4 hours. Heterodimer formation observed with GDC-0879 alone is further increased upon co-administration of 1 µM MEK inhibitor Mek-i A. Bottom: CRaf IP kinase activity assays from lysates of cells treated as above. CRaf was immunoprecipitated from treated cells and kinase activity towards recombinant MEK was tested in vitro. Co-administration of Mek-i A with GDC0879 resulted in a dose-dependent increase in maximal CRaf kinase activity across all GDC0879 doses. For the CRaf kinase activity assays, relative phospho-MEK levels measured with MSD pMEK ELISA assay are shown. (**E**) Four-day proliferation curves of Ras^WT^/Raf^WT^ cells (SW48 colon carcinoma) upon combination treatment of GDC-0879 with increasing concentrations of Mek-i A show induction of viable cell counts compared to MEK inhibitor treatment alone in a dose dependent manner with a maximal effect at co-administration of 1 µM of GDC0879. (**F**) Model describing the localization effect of Raf, Mek and Erk inhibitors on Raf cellular localization. Ras activation causes CRaf recruitment to the PM, priming of CRaf or inhibition of negative downstream feedback increase CRaf targeting to the PM. Combination of priming and feedback inhibition additively promote CRaf PM targeting.

Based on our priming studies described above, we hypothesized that since mechanisms for Raf PM targeting differ for Raf inhibitors versus Mek/Erk inhibitors, their combination may show an additive effect on CRaf PM levels. To test this hypothesis, we treated RasG12D/CRaf expressing cells with each Mek and Erk inhibitor in the presence of different Raf inhibitors at ∼EC_50_ doses. Indeed, the Mek inhibitors and the Erk inhibitor all showed an additive effect on CRaf PM targeting in combination with each of the Raf inhibitors as indicated by an increase in maximal effect without a large shift in EC_50_ (Fig. 4a–c) [32]. The additive effect between Raf and Mek inhibitors on CRaf PM translocation was observed when measuring CRaf/BRaf hetero-dimerization and CRaf kinase activation induced by GDC0879 in the KRasG12D/CRaf cells (Fig. 4d) as well as in H226 NSCLC cells where only endogenous proteins are expressed (Figure S8). To determine whether this additive effect on CRaf activity may have functional downstream consequences, we treated Ras/Raf^WT^ tumor cells with GDC0879 +/− Mek-i A and measured cell proliferation. Combination with GDC0879 blocked the anti-proliferative effect of Mek-i A administered at low doses (Fig. 4e). These results suggest that PM translocation of CRaf is a relevant marker of pathway activation, leading to cellular hyperproliferation downstream of Raf inhibitor treatment of BRaf^WT^ cells.

Next, we tested combinations of Raf inhibitors, since each of them has distinct binding modes to Raf [9]. Interestingly, the Raf inhibitor AZD628 has synergistic activity in combination with the Raf inhibitors GDC0879 and PLX4720 as indicated by a large decrease in EC_50_ [32], whereas the combination of GDC0879 and PLX4720 did not have a synergistic effect (Figure S9a–c). Synergy between AZD628 and GDC0879 or PLX4720 was also seen when looking at CRaf-BRaf heterodimerization and CRaf kinase activity (Fig. S9 d and e). The difference between Raf inhibitor combination effects might be due to the preferred Raf binding conformation for each compound: AZD628 binds to the inactive conformation (DFG-out) of Craf, whereas both GDC0879 and PLX4720 bind to the active CRaf conformation (DFG-in) [9], with PLX4720 causing a c-helix shift that may block BRaf:CRaf heterodimer formation, as observed in our current study. Such detailed analysis for structurally dissimilar inhibitors of a common target may provide further insight into their mechanistic differences (Fig. 4f).

### Effect of MAPK pathway inhibitors on RBDCRD cell line

**Figure 5:**
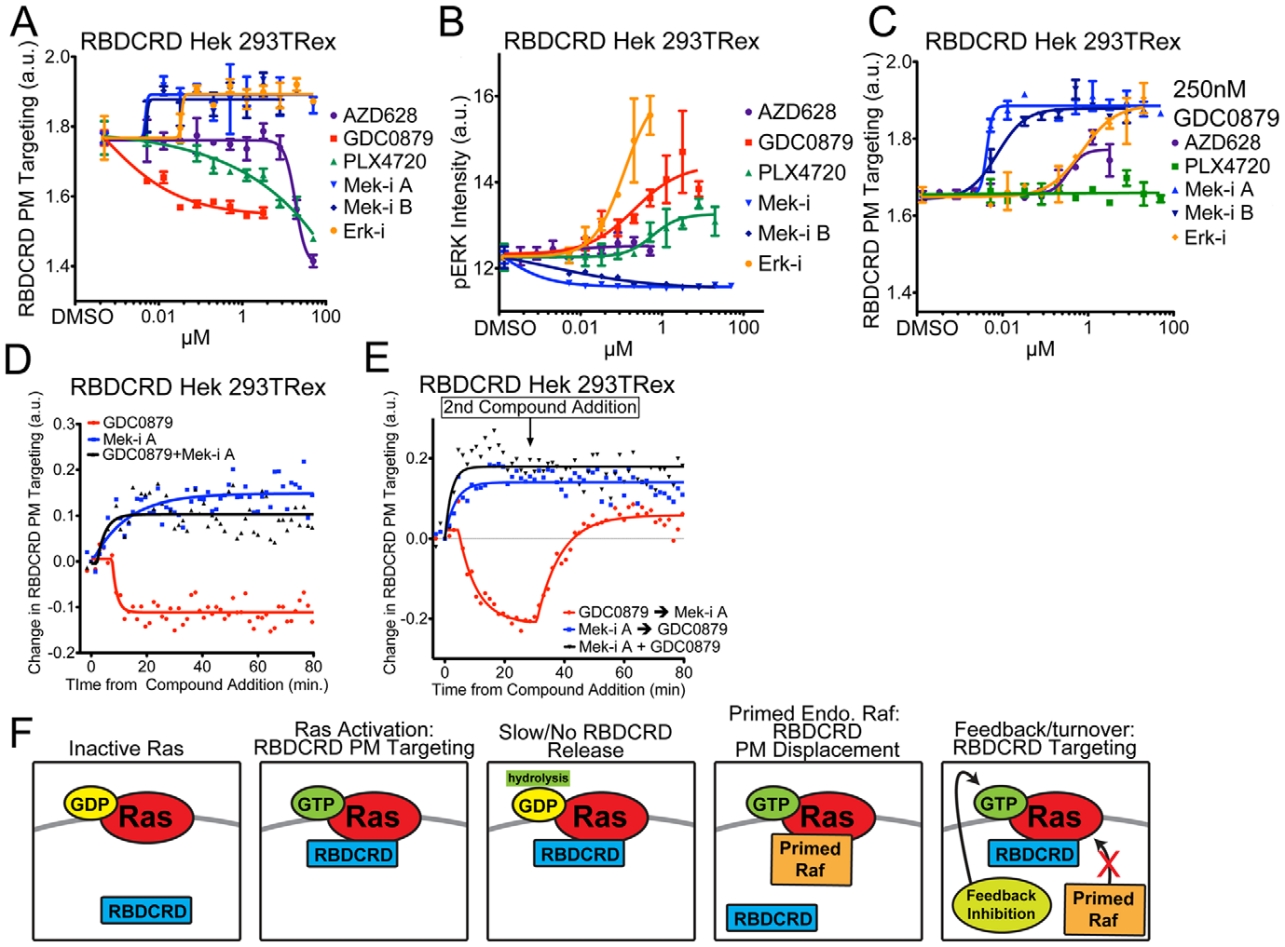
RBDCRD response to MAP-kinase inhibitors. (**A**) RBDCRD PM targeting was measured in Venus-RBDCRD stable cell line after 4 hr treatment with AZD628, GDC0879, PLX4720, Mek-i A, Mek-i B, or Erk-i. (**B**) phospho-ERK intensity was measured by immuno-fluorescence in cells treated as in (A). (**C**) RBDCRD PM targeting was measured in cells treated with a constant dose of 250 nM GDC0879 with a dose curve of the other small molecule inhibitor, showing the dominant effect of Mek and Erk inhibitor-induced targeting. (**D**) Time-lapse experiments show the kinetics of RBDCRD PM targeting and displacement upon treatment with 250 nM GDC0879, 20 nM Mek-i A or the combination of both. (**E**) Time-lapse experiment as in (D) where GDC0879 and Mek-i A were added sequentially with a 28.5 min. time gap between compound additions. (**F**) Schematic model describing RBDCRD cellular behavior in the presence of MAPK inhibitors.

To investigate how endogenous Ras activation is affected by various MAPK pathway inhibitors, different inhibitor classes were administered to HEK 293 TRex cells expressing RBDCRD. These cells should respond to increased RasGTP levels but not to Raf inhibitor priming, since RBDCRD lacks the Raf ATP binding pocket (Fig. 5a). As predicted, both Mek and Erk inhibitors induced an increase in PM targeting of RBDCRD consistent with the release of negative feedback and an increase in RasGTP levels. Surprisingly, the three Raf inhibitors caused a decrease in RBDCRD PM targeting, suggesting that endogenous Raf, once primed by Raf inhibitors, can displace RBDCRD from Ras at the PM. Consistent with this model, pERK levels in the RBDCRD cell line increased with the addition of Raf inhibitors but not Mek inhibitors (Fig. 5b). However, when a Raf inhibitor was combined with either Mek or Erk inhibitors, the displacement of RBDCRD seen with Raf inhibitor alone was abolished. Instead, RBDCRD was recruited to the membrane to a degree similar to that induced by Mek and Erk inhibitors alone, suggesting that Ras activation may lock RBDCRD at the PM before priming-induced displacement of RBDCRD can occur (Fig. 5c).

We next used kinetic experiments to further characterize the effects of negative feedback release vs. priming in the RBDCRD cell line and understand the interaction of RBDCRD with RasGTP. Addition of Mek-i A caused recruitment of RBDCRD to the PM within 1.5 min., whereas the onset of RBDCRD displacement after addition of GDC0879 occurred after 7.5 min. (Fig. 5d). When GDC0879 and Mek-i A were combined, RBDCRD recruitment was similar to that of Mek-i A alone. Furthermore, when GDC0879 and Mek-i A were added sequentially during time-lapse imaging with a 28.5 minute interval between compound additions, the initial displacement of RBDCRD from the PM was fully recovered upon Mek-i A addition (Fig. 5E). Based on our findings, we propose a model whereby RBDCRD does not readily release from Ras upon GTP hydrolysis. This idea is supported by the lack of reversibility of RBDCRD PM targeting upon EGF stimulation (Fig. 2e) as well as *in vitro* biophysical data [33]. Notably, recent work has demonstrated that RBDCRD binding to WT Ras or RasG12V does not effect rates of hydrolysis [34]. A lack of release from RasGDP would also explain why RBDCRD is so highly enriched at the PM in cells with endogenous Ras, where only a small fraction of Ras is GTP-bound at any given time. Over time, the stochastic activation of Ras should lead to a large accumulation of RBDCRD at the PM. Supporting this idea, when RBDCRD PM targeting is measured upon RBDCRD induction in the HEK 293 stable cell line, maximal PM targeting is seen only after 10 h of induction (Figure S5c). Taken together, our study of RBDCRD reveals a novel mechanistic model to explain the unique cellular behavior of this fragment of CRaf (Fig. 5f).

### Visualizing the MAPK pathway

**Figure 6:**
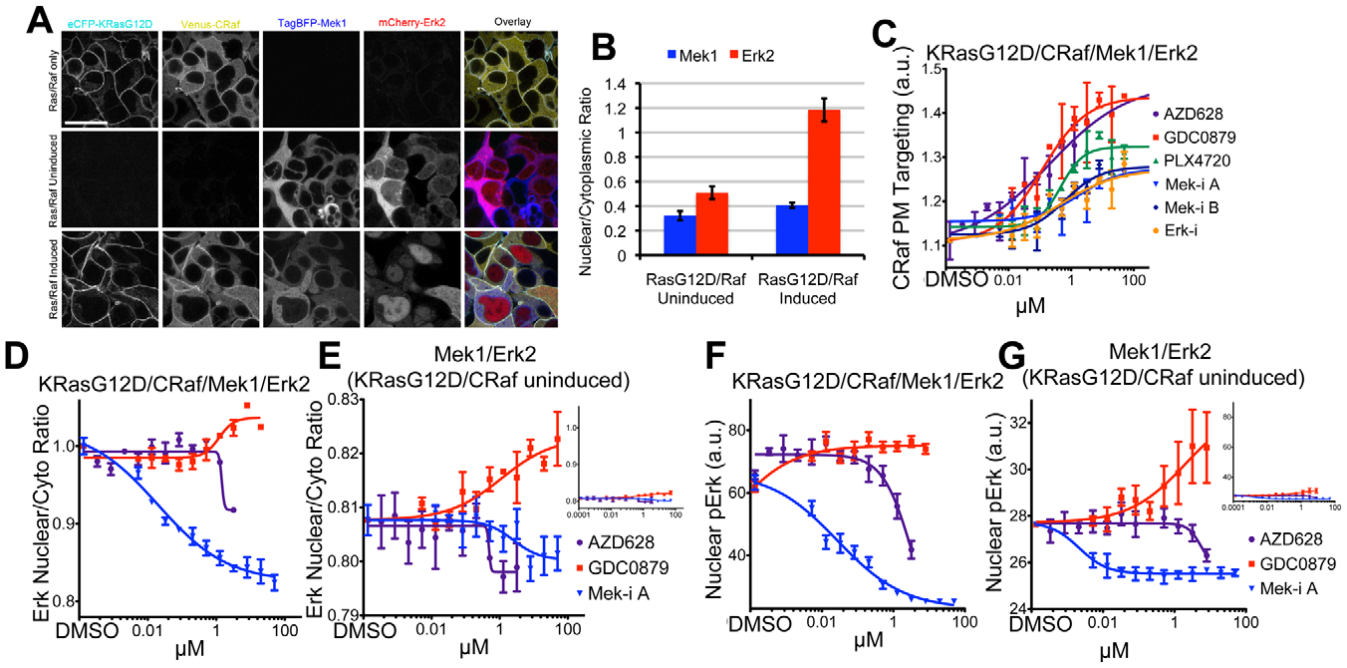
Visualization of the labeled MAPK pathway. (**A**) Example images show induced parental KRasG12D/CRaf cell line, induced and uninduced 4-color cell line where TagBFP-Mek1 and mCherry-Erk2 are constitutively expressed. Scale bar is 20 µm and applies to all panels. (**B**) Nuclear localization of TagBFP-Mek1 and mCherry-Erk2 was measured in MAPK cell line where eCFP-KRasG12D and Venus-CRaf were either induced or uninduced. (**C**) MAPK cell line was dosed with Raf, Mek and Erk inhbitors, CRaf PM localization was measured. (**D**) The nuclear/cytoplasmic ratio of mCherry-Erk1 was measured in MAPK cell line where KRasG12D and CRaf expression were induced. (**E**) The nuclear/cytoplasmic ratio of mCherry-Erk1 was measured in the MAPK cell line where KRasG12D and CRaf were not induced. (**F**) Nuclear pERK was measured in the HEK 293 KRasG12D/CRaf/Mek1/Erk2 cell line by immuno-fluorescence 5hrs after inhibitor treatment. (**G**) Nuclear pERK was measured as in (F) in the HEK 293 cell line expressing TagBFP-Mek1 and mCherry-Erk2 where expression of KRasG12D and CRaf is not induced.

Finally, since KRas and CRaf are expressed at higher than endogenous levels in the inducible HEK293 cell line (Figure S3), we wanted to increase the expression levels of Mek and Erk to determine whether feedback signaling to Raf was affected. TagBFP-Mek1 and mCherry-Erk2 were added to the Ras/Raf cell line using the pIRES3 vector system. Mek1 and Erk2 were both primarily localized in the cytoplasm in the absence of eCFP-KRasG12D and Venus-CRaf induction. Upon KRasG12D/CRaf induction, Erk2 accumulated in the nucleus and pERK levels increased, consistent with the expected induction of activity (Fig. 6a and b and Figure S3) [35,36]. As expected, both Raf inhibitor priming and Mek/Erk inhibitor negative feedback release caused CRaf targeting to the PM in a dose-dependent manner in the RasG12D/CRaf/Mek1/Erk2 cell line (Fig. 6c). No changes were detected in the cytosolic localization of TagBFP-Mek1 (data not shown). We next looked at the targeting of Erk to the nucleus by measuring cytoplasmic versus nuclear intensities of mCherry-Erk2 and calculating the nuclear/cytoplasmic ratio. These ratios were measured at the individual cell level since the expression level of mCherry-Erk2 was variable. Mek-i A caused a decrease in nuclear Erk as expected, whereas interestingly the Raf inhibitor GDC0879 caused a subtle but reproducible increase in nuclear Erk levels (Fig. 6d). At high doses the Raf inhibitor AZD628 caused a decrease in nuclear Erk, consistent with its biochemical potency on CRaf [9]. Since the MAPK pathway is highly activated upon the induction of KRasG12D and CRaf (Figure S3), we also looked at nuclear Erk levels in the KRasG12D/CRaf/Mek1/Erk2 cell line in the absence of KRasG12D and CRaf induction. In this setting, the decreases in nuclear Erk seen with Mek-i A and AZD628 treatments were subtle, whereas the increase caused by GDC0879 was more dramatic than in the RasG12D/CRaf induced cells (Fig. 6e). Since basal pERK levels were lower in uninduced cells, the activating effect of GDC0879 on pERK became more pronounced (Fig. 6f and g and Figure S10). Since pERK levels correlated well with Erk2 nuclear localization in our studies, we confirm previous reports that nuclear Erk acts as a reliable live-cell readout of Erk activation [37]. Furthermore, our data suggest that the KRasG12D/CRaf/Mek1/Erk2 cell line can be used to identify both suppressors and activators of Erk activity.

## Discussion

Live cell microscopy assays are ideally suited for studying the spatial and temporal aspects of cellular signaling. While quantitative, high-throughput time-lapse microscopy has most commonly been applied in the context of siRNA screens to identify modulators of the cell cycle [38], our work highlights the utility of these approaches to visualize compound-mediated perturbations of pathway dynamics in a commonly mutated oncogenic signaling pathway. We report advances in cell line engineering, image acquisition and analysis that enabled us to carry out a high-throughput investigation of a panel of inhibitors with diverse specificity, potency, binding, and mechanisms of action and perform systematic and comprehensive analysis of their impact on pathway activity individually and in combination within living cells.

We observed that inhibitors of MEK and ERK promote pathway activation through release of negative feedback, consistent with previous data. Time-lapse experiments demonstrated that the dynamics of pathway activation via the multistep process of negative feedback release are slower and more stoichiometrically regulated than direct pathway activation by Raf inhibitor priming. Combination of Raf inhibitors with MEK or ERK inhibitors led to an additive promotion of MAPK pathway activation through the combined effects of negative feedback release and priming. Furthermore, combination of two Raf inhibitors with different modes of binding to Raf, e.g., AZD628 combined with either GDC0879 or PLX4720, resulted in a synergistic increase in pathway activation, whereas combination of inhibitors with similar binding modes, i.e., GDC0879 and PLX4720, did not result in synergy. In future studies, it would be interesting to investigate the localization dynamics and MAPK activity using reporters of BRafV600E, as the Raf inhibitors used in this study have been shown to act to block V600E activity [11]. In sum, these observations illustrate the potential of this approach to systematically screen inhibitor combinations with diverse specificities, potencies and binding modes to exclude those with undisirable effects and to identify those that are most likely to offer maximum therapeutic benefit.

Regarding RBDCRD, a fragment of CRaf that has been reported to be a sensor of endogenous activated Ras [13], a claim that has been disputed by more recent work [23], we confirmed its ability to sense endogenous RasGTP in several cell types and generated an RBDCRD reporter cell line for use in subsequent quantitative high throughput analysis. Subjecting this cell line to inhibitors that promote priming or release negative feedback or combinations thereof allowed us to detect differential effects of feedback and priming mediated by endogenous proteins and supported a refined model for the mechanism of RBDCRD binding to and release from Ras. Our data are consistent with a model in which the RBDCRD off-rate from Ras is much slower than that of full-length CRaf, which can reconcile the ability of the RBDCRD fragment to detect endogenous RasGTP levels driven by the release of negative feedback from the membrane displacement of RBDCRD seen with inhibitor-induced Raf priming.

In the last several decades, drug discovery has yielded diverse strategies for targeting dysregulated oncogenic signaling pathways. Although the relevance of the intracellular environment to the activity of these pathways is becoming increasingly appreciated [39], in the absence of appropriate cell-based assays, typical drug discovery efforts are predicated on *in vitro* mechanistic biochemical assays followed by non-mechanistic cell viability assays that do not offer insight into the subtleties of drug mechanism of action in live cells. An inherent risk in this strategy is highlighted by the observation cited above that Raf inhibitors prime Raf in certain cellular contexts, thus promoting therapeutically undesirable MAPK pathway activation. Our data indicate that mechanistic cellular assays that can detect inappropriate pathway activation in a cellular environment and are amenable to high-throughput analysis can be an important tool in effectively prioritizing the development of safe and efficacious targeted drugs and in identifying productive therapy combinations.

## Materials and Methods

### Molecular biology

Fluorescent fusion proteins were generated using two-insert multisite Gateway® cloning (Invitrogen) where one insert contained the fluorescent protein tag and the other contained gene of study, these constructs where inserted into pCDNA-DEST47 with a stop codon to prevent translation of the c-terminal GFP. An IRES element was inserted into the middle of the multiple cloning site of pCDNA5/FRT/TO (pFIT), fluorescent-fusion genes were then cloned into the pFIT vector on both sides of the IRES using In-Fusion PCR cloning (Clontech). For the construction of the eCFP-KRasG12D Venus-CRaf TagBFP-Mek1 mCherry-Erk2 cell line, the Gateway® cassette was added to the pIRES3 vectors (Clontech) using the Gateway® conversion system (Invitrogen) and the fluorescent fusion constructs for Mek1 are Erk2 were inserted using multisite Gateway.

### Cell line engineering and culturing

HEK 293T (ATCC™) were grown in DMEM-F12 50∶50 media with 10% fetal bovine serum (FBS) and 1X Glutamax (Invitrogen™), HEK 293 TRex cells (Invitrogen™) were cultured in DMEM-F12 50∶50 with 10% Tet-free FBS and 1X Glutamax. NCI-H727 (ATCC™) were cultured in RPMI with 10% FBS and 1X Glutamax, Hec1A [25] cells were grown in McCoy's media with 10% FBS and 1X Glutamax. All cell lines were cultured at 37°C with 5% C0_2_. Transient transfections were carried out in HEK293T, NCI-H727 and Hec1A cells were done using Lipofectamine2000 (Invitrogen™) using the manufactures suggested protocol. T-REx™ cell lines were made following the manufactures protocol (Invitrogen™). pIRES3 vectors were transfected into T-REx™ stable cell lines, drug selection [100 µg/ml Geneticin or 500 ng/ml puromycin] was added 2 days after trasfection. For microscopy-based assays, cells were plated at 13,000–22,000 cells per well in coverslip-bottom 384 well plates (Aurora Biotechnologies). For experiments involving the HEK 293 T-REx™ cell lines, cells were induced with 1 µg/ml Doxycycline 24 hrs after cell plating and 20–30 before imaging. Compounds were added 2.5–4 hrs prior to imaging for end-point assays or after three frames of acquisition for time-lapse studies.

### Microscopy

384 well plates were imaged with a Nikon Ti-perfect focus inverted micrscope with an A1R resonant spectral confocal system with temperature and environmental (37°C 5% CO_2_) control. Automated macros that facilitated automated imaging of the inner 308 wells of the plate were used for acquistion. Most images were acquired with a CFI Plan Fluor 40X oil immersion objective (NA: 1.3, Nikon), full plate acquisition was possible by first spreading 37°C immersion oil (Cargille) on the plate bottom with an ink roller. Ti-Perfect focus functionality was used to ensure proper oil interface was maintained between plate bottom and objective, with a custom-built macro used to correct loss in interface (Austin Blanco, Technical Instruments). Low-resolution images of pERK experiments were acquired with a CFI Plan Apo VC 20X objective (NA: 0.75, Nikon).

### Image and Data Analysis

Custom image analysis programs were written in MATLAB® (MathWorks®) to segment cells into PM, cytoplasm/nuclear compartments and subsequently measure fluorescent intensity within each of these compartments (see Materials S1). Cellular centroids were identified by either the nuclear absence of Venus-CRaf, the absence mCherry-PMem signal in the nuclear and cytoplasmic space or by the presence of fluorescently labeled H2B. For PM targeting measurements, 360 pixel ratios at the PM and adjacent cytoplasm were measured per cell, 2 fields of ∼200–400 cells per field were analyzed per condition. Experimetns were repeated as three biological replicates and data from each expereiment was combined to graphing. For nuclear ERK targeting the nuclear/cytoplasmic ratio of mCherry-Erk2 was measured in individual cells and then averaged per field. Nuclear and cytoplasmic pERK intensity were measured in bulk per image.

Numerical data generated from image analysis was plotted and fitted using Excel (Microsoft) and Prism (Graph Pad). Dose-curve experiments were fitted with a variable slope sigmoid curve and time-lapse experiments were fit to a single exponential. All error bars represent standard error and p-values were calculated using T-test.

### Biochemical Experiments

Biochemical experiments were conducted as described in Hatzivassiliou et al. [9].

## Supporting Information

Figure S1 **Membrane targeting of CRaf and CRaf fragments with KRas.** (**A**) 293T cells were transfected with either eCFP-KRasWT or eCFP-KRasG12D together with Venus- CRaf RBD, Venus-FL-CRaf or Venus-CRaf RBDCRD and mCherry-H2B. Scale bar is 20µm and applies to all panels. (**B**) PM targeting of transient transfection experiment described in (A) was measured, along with cells transfected with eCFP-KRasS17N, a dominant negative mutant, and eCFP-CAAX as a PM reporter.
(TIFF)

Figure S2 **Akt1 stable cell line show subtle serum-dependent PM targeting.** (**A**) A stable 293 T-REx™ cell line expressing Venus-Akt1 and eCFP-PMem (CAAX motife) was generated, example images of cells starved of serum for 12hrs or with 10% FBS are shown. Scale bar is 20µm and applies to all panels. (**B**) PM targeting of Venus-Akt1 was measured +/- serum using automated PM targeting program, p = 0.016.
(TIFF)

Figure S3 **Western blot analysis of 293 T-REx™ stable cell lines.** Cells were either cultured without doxycycline or induced with 100ng/ml doxycycline for 24hrs before harvesting cell lysates. (**A**) Expression of both Venus-fusion and eCFP-fusion constructs were detected using an anti-GFP antibody, Venus-RBDCRD migrated faster than eCFP-KRas and the larger Venus-CRaf. (**B**) Both endogenous and Venus-CRaf were detected with anti-CRaf antibody, showing strong Tet-repression in the absence of Dox. (**C**) Cell lysates expressing both endogenous Ras and eCFP-KRas were probed with an anti-KRas antibody.
(TIFF)

Figure S4 **RBDCRD PM Targeting.** (**A**) HEK 293T cells were transfected with Venus-RBDCRD and cells with varying expression of were imaged (top row), when intensites were normalized (bottom row) inverse correlation with PM targeting and expression level was seen suggesting a limited number of RBDCRD binding sites at the PM. Scale bar is 10µm and applies to all panels. (**B**) HEK 293 T-REx™ Venus-RBDCRD, mCherry-PMem stable cell line was generated, and images were acquired for cells grown with serum, cells which were starved of serum for 12hrs and for cells transiently transfected with the dominant negative eCFP-KRasS17N. (**C**) RBDCRD PM targeting was measured for experiment described in (B). Scale bar is 20µm and applies to all panels. (**D**) Example images for EGF stimulation of HEK 293T cells transfected with Venus-RBDCRD quantified in Figure 2E. Scale bar is 20µm and applies to all panels.
(TIFF)

Figure S5 **MCP110 disrupts RBDCRD PM targeting.** (**A**) HEK 293 T-REx™ Venus-RBDCRD cell line was treated with MCP110 and images were acquired after 2.5hrs. Scale bar is 20µm and applies to all panels. (**B**) Venus-RBDCRD PM targeting was measured for cells dosed with MCP110. (**C**) HEK 293 T-REx™ Venus-RBDCRD cell line was induced with doxycycline and imaged every 30min, PM/cytoplasmic ratios of RBDCRD were then measured.
(TIFF)

Figure S6 **Dose dependent MAPK pathway inhibition by MEK (Mek-i A, Mek-I B) and Erk-i inhibitors (panel A) leads to release of negative feedback MAPK phosphorylation site on CRaf (panel B).** Cells were treated with indicated inhibitor concentrations for 4 hours and lysates probed by immunoblot for pRSK (T359/363) and pCRaf(S289/296/301) levels (bottom). Curves represent quantitation of WB with Typhoon and curve fitting using Prism. (C,D) Western blot images of (A) and (B) respectively.
(TIFF)

Figure S7 **CRaf PM targeting is rapid with Mek-i A in KRasG12D/CRaf/Mek1/Erk2 cell line.** MAPK cell line was imaged with time-lapse micrscopy and CRaf PM targeting was measured with the addition of 10µM GDC0879 or 10µM Mek-i A, images were acquired every 2min.
(TIFF)

Figure S8 **Additivity in targeting CRaf to the plasma membrane through combined priming by RAF inhibitors and negative feedback release by MEK inhibitors in H226 (KRas^WT^/CRaf^WT^) NSCLC cells.** Targeting CRaf to the plasma membrane upon combined priming by RAF inhibitors and negative feedback release by MEK inhibitors in H226 (KRas^WT^/CRaf^WT^) NSCLC cells. (**A**) Top: Immunoblot of Braf:Craf heterodimers in H226 cells treated with GDC-0879 (0.1, 1, 10 µM) for 24 hours in cells pretreated for 1 hour with either DMSO or 1 µM Mek-i A. Heterodimer formation observed with Raf inhibitors alone is further increased upon co-administration of 1 μM MEK inhibitor Mek-i A. Bottom: Western blot of lysates from cells treated as above with indicated antibodies showing induction of pCRaf S338 and phospho-MEK levels. (**B**) CRaf IP kinase activity assays from lysates of cells treated as above. CRaf was immunoprecipitated from treated cells and kinase activity towards recombinant MEK was tested in vitro. Co-administration of Mek-i A with GDC-0879 resulted in a dose-dependent increase in maximal CRaf kinase activity across all Raf inhibitor doses. For the CRaf kinase activity assays, shown are relative phospho-MEK levels measured with MSD pMEK ELISA assay.
(TIFF)

Figure S9 **The DFG-out Raf inhibitor AZD-628 synergizes with the DFG-in inhibitors GDC-0879 and PLX4720 to potentiate CRaf activation in KRasG12D/CRaf 293 T-REx™ cells. Antagonism is observed between DFG-in Raf inhibitor GDC-0879 and DFG-in/c-helix shift PLX4720.** (**A**) KRasG12D/CRaf HEK 293 T-REx™ cells were dosed with varying concentrations of AZD628 and constant dose of 2µM PLX4720 or 300nM GDC0879 and CRaf PM targeting was measured after 4hrs. (**B**) KRasG12D/CRaf HEK 293 T-REx™ cells were dosed with varying concentrations of GDC0879 and constant dose of 2µM PLX4720 or 12nM AZD628 and CRaf PM targeting was measured after 4hrs. (**C**) KRasG12D/CRaf HEK 293 T-REx™ cells were dosed with varying concentrations of PLX4720 and constant dose of 12nM AZD628 or 300nM GDC0879 and CRaf PM targeting was measured after 4hrs. (**D**) Top: Immunoblot of immunoprecipitated (IP) Craf shows Braf:Craf heterodimers in cells treated with AZD-628 (0.001 and 0.01) for 4 hours. Heterodimers are further induced in the presence of 50 nM GDC-0879 (middle panel) and reduced in the presence of 1 μM PLX4720, which disrupts heterodimer formation due to induction of a c-helix shift in BRaf and CRaf. Bottom: CRaf IP kinase activity assays from lysates of KRasG12D/Craf 293 T-REx™ cells treated with 0.001 and 0.01 µM AZD-628 for 4 hours. CRaf was immunoprecipitated from treated cells and kinase activity towards recombinant MEK was tested in vitro. (**E**) Top: Immunoblot of BRaf:CRaf heterodimers in cells treated with PLX4720 (0.1, 1, 10) for 4 hours. Weak heterodimer formation observed with PLX4720 alone, compared to treatment with 300 nM GDC-0879 alone (5th lane, DMSO/GDC-0879 panel). Increasing amounts of PLX4720 further destabilize BRaf:CRaf heterodimers due to distinct binding mode of PLX4720. Bottom: CRaf IP kinase activity assays from lysates of cells treated with PLX4720 (0.1, 1, 10) for 4 hours. CRaf was immunoprecipitated from treated cells and kinase activity towards recombinant MEK was tested *in vitro*. Adding PLX4720 to GDC-0879 treated cells leads to a dose-dependent decrase in maximal CRaf kinase activity. For all Craf kinase activity assays, relative phospho-MEK levels measured with an MSD pMEK ELISA assay are shown.
(TIFF)

Figure S10 **Images of mCherry-Erk1 localization and phospho-ERK.** (**A**) Example images of mCherry-Erk2 sub-cellular localization, imaged 4hrs after inhibitor treatments with KRasG12D and CRaf induced. Scale bar is 20µm and applies to all panels. (**B**) Example images of mCherry-Erk2 sub-cellular localization, imaged 4hrs after inhibitor treatments with KRasG12D and CRaf uninduced. Scale bar is 20µm and applies to all panels. (**C**) Example images of pERK immuno-fluorescence, stained and imaged 5hrs after inhibitor treatments with KRas and CRaf induced. Scale bar is 20µm and applies to all panels. (**D**) Example images of pERK immuno-fluorescence, stained and imaged 5hrs after inhibitor treatments with KRas and CRaf uninduced. Scale bar is 20µm and applies to all panels.
(TIFF)

Materials S1 **Matlab Programs.**
(DOCX)
