# Supplementary figures and images for: Live-Cell Microscopy Reveals Small Molecule Inhibitor Effects on MAPK Pathway Dynamics
Source: PLoS One. 2011 Aug 4;6(8):e22607. doi: 10.1371/journal.pone.0022607 (PMC3150364; doi:10.1371/journal.pone.0022607)

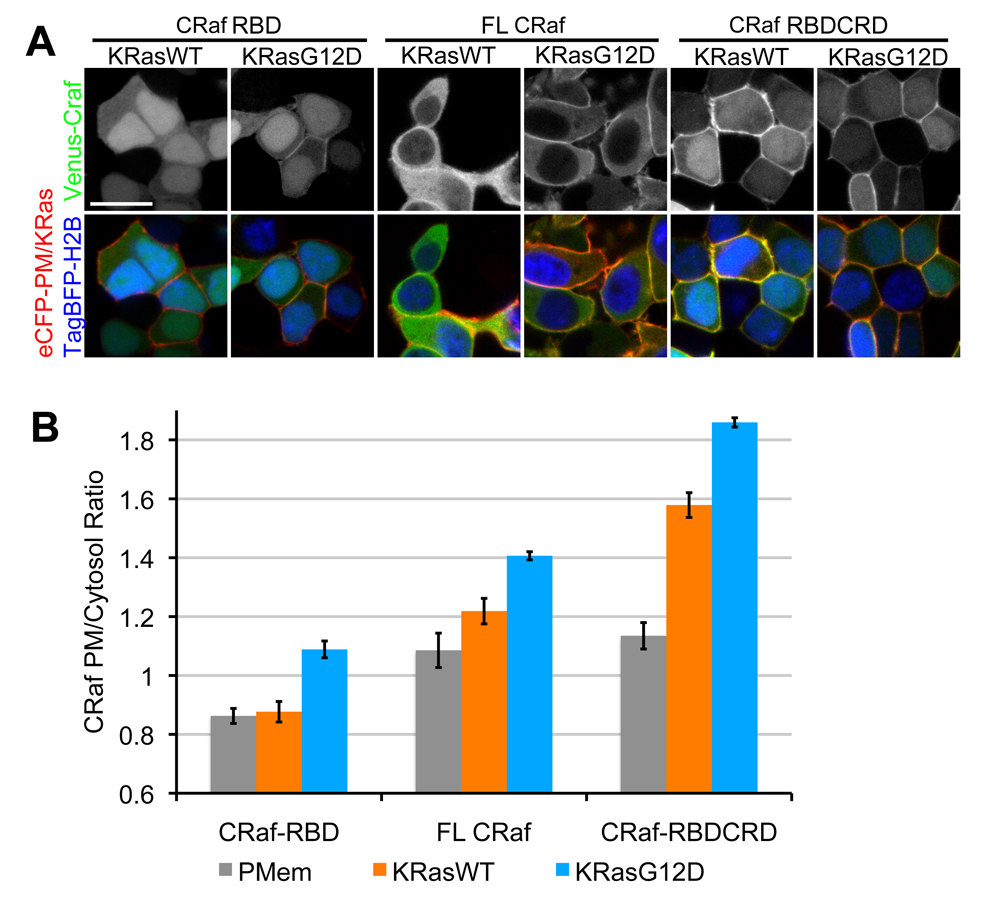

Supplement: Figure S1 — Membrane targeting of CRaf and CRaf fragments with KRas. (A) 293T cells were transfected with either eCFP-KRasWT or eCFP-KRasG12D together with Venus- CRaf RBD, Venus-FL-CRaf or Venus-CRaf RBDCRD and mCherry-H2B. Scale bar is 20µm and applies to all panels. (B) PM targeting of transient transfection experiment described in (A) was measured, along with cells transfected with eCFP-KRasS17N, a dominant negative mutant, and eCFP-CAAX as a PM reporter. (TIFF) [file pone.0022607.s001.tif]

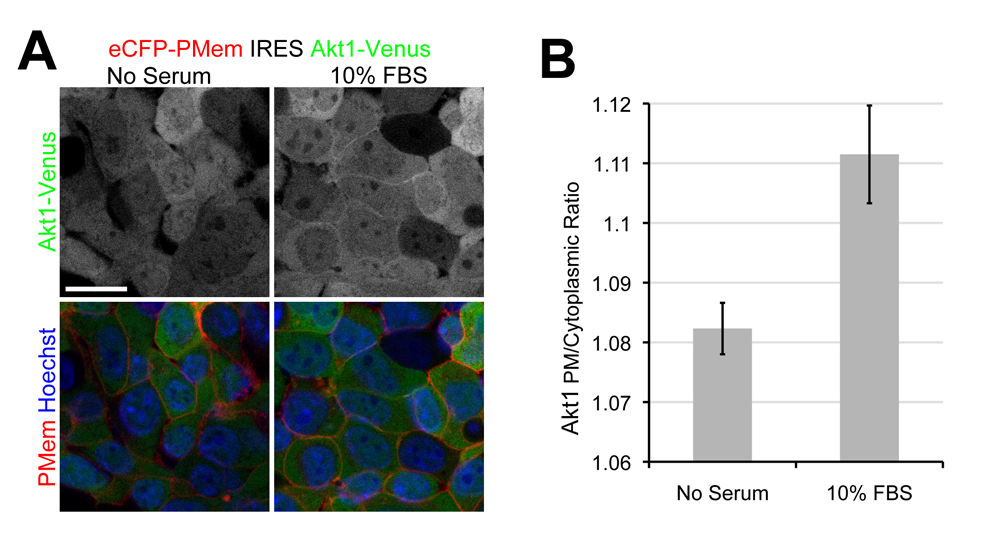

Supplement: Figure S2 — Akt1 stable cell line show subtle serum-dependent PM targeting. (A) A stable 293 T-REx™ cell line expressing Venus-Akt1 and eCFP-PMem (CAAX motife) was generated, example images of cells starved of serum for 12hrs or with 10% FBS are shown. Scale bar is 20µm and applies to all panels. (B) PM targeting of Venus-Akt1 was measured +/- serum using automated PM targeting program, p = 0.016. (TIFF) [file pone.0022607.s002.tif]

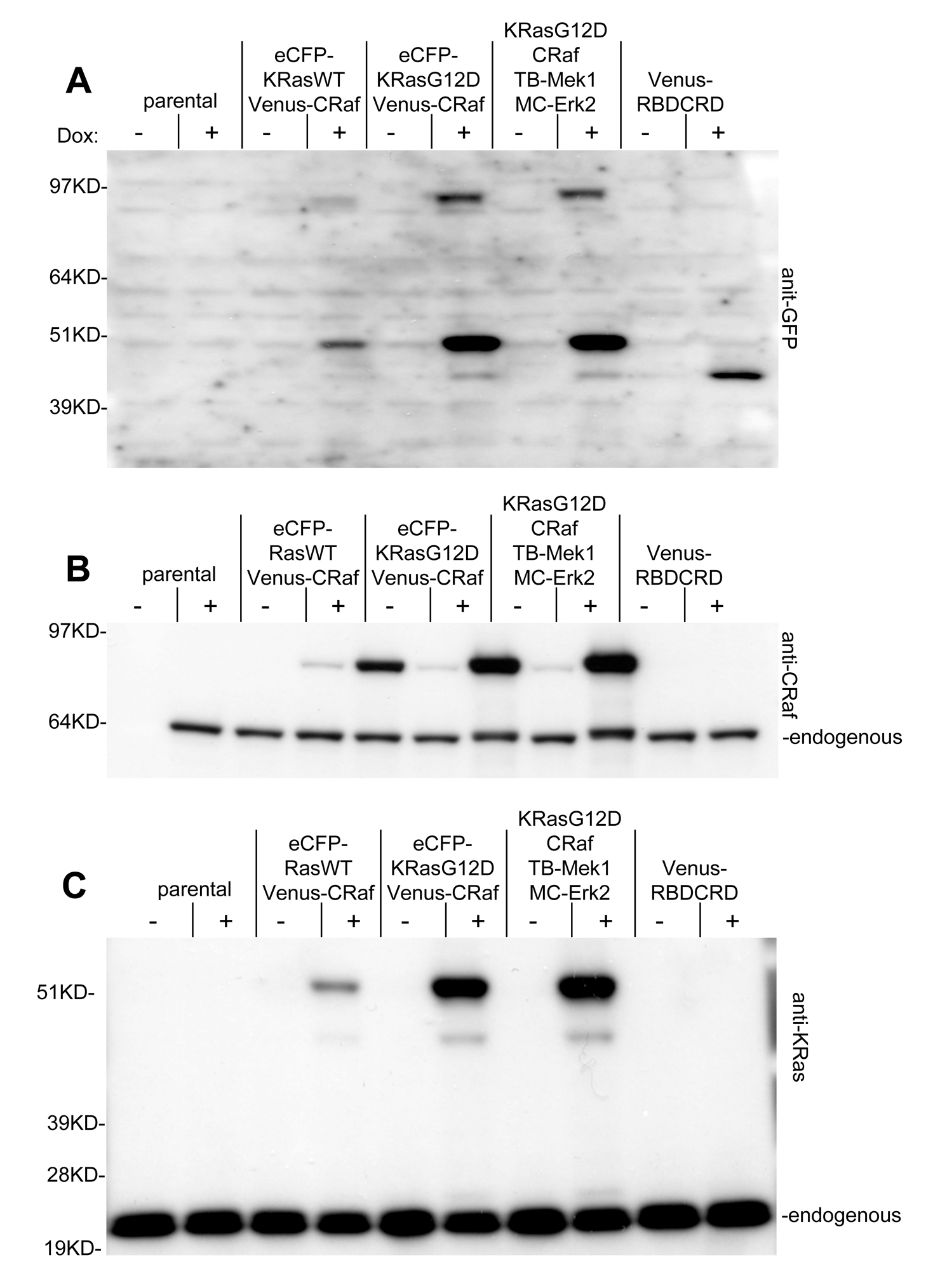

Supplement: Figure S3 — Western blot analysis of 293 T-REx™ stable cell lines. Cells were either cultured without doxycycline or induced with 100ng/ml doxycycline for 24hrs before harvesting cell lysates. (A) Expression of both Venus-fusion and eCFP-fusion constructs were detected using an anti-GFP antibody, Venus-RBDCRD migrated faster than eCFP-KRas and the larger Venus-CRaf. (B) Both endogenous and Venus-CRaf were detected with anti-CRaf antibody, showing strong Tet-repression in the absence of Dox. (C) Cell lysates expressing both endogenous Ras and eCFP-KRas were probed with an anti-KRas antibody. (TIFF) [file pone.0022607.s003.tif]

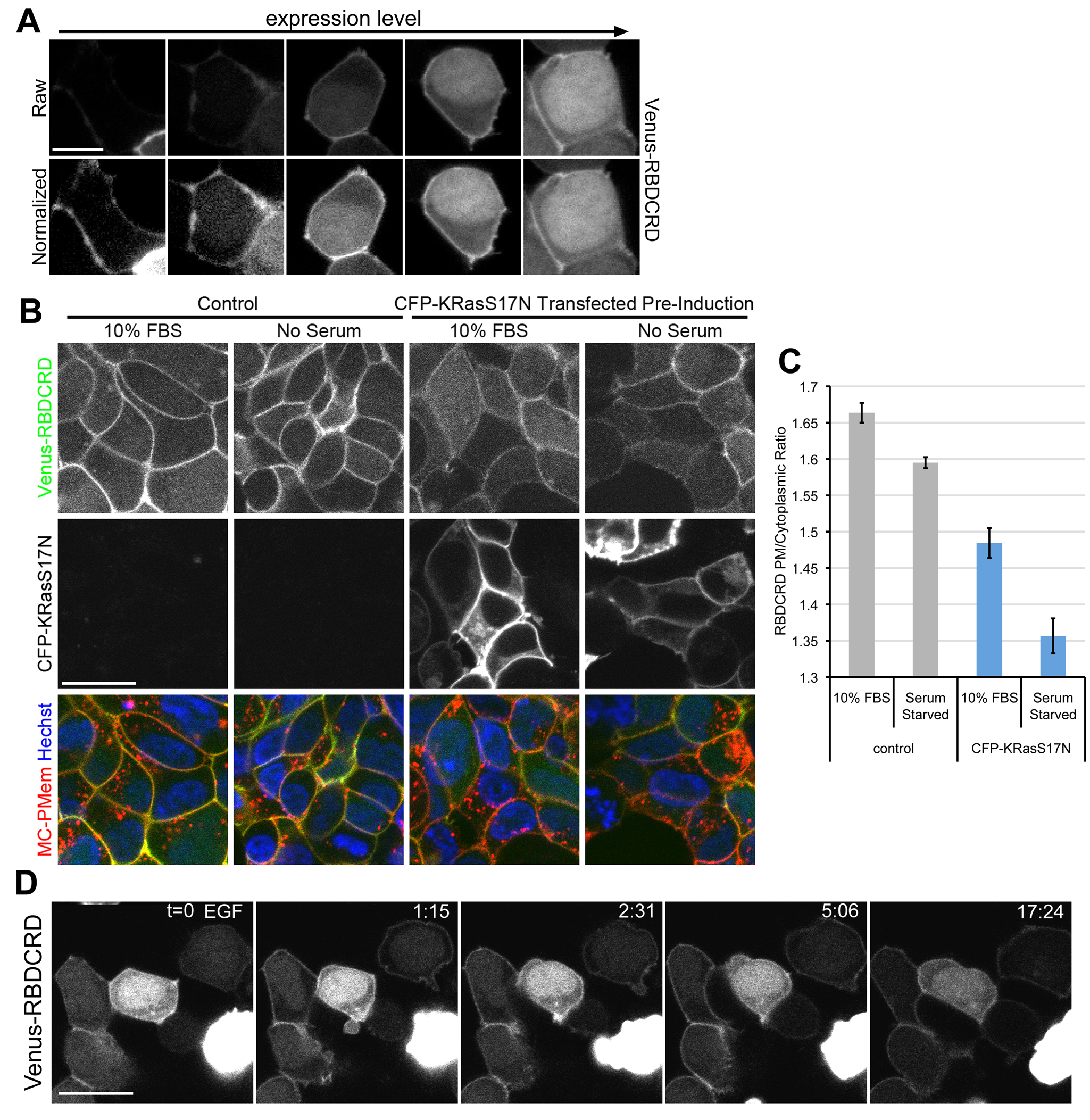

Supplement: Figure S4 — RBDCRD PM Targeting. (A) HEK 293T cells were transfected with Venus-RBDCRD and cells with varying expression of were imaged (top row), when intensites were normalized (bottom row) inverse correlation with PM targeting and expression level was seen suggesting a limited number of RBDCRD binding sites at the PM. Scale bar is 10µm and applies to all panels. (B) HEK 293 T-REx™ Venus-RBDCRD, mCherry-PMem stable cell line was generated, and images were acquired for cells grown with serum, cells which were starved of serum for 12hrs and for cells transiently transfected with the dominant negative eCFP-KRasS17N. (C) RBDCRD PM targeting was measured for experiment described in (B). Scale bar is 20µm and applies to all panels. (D) Example images for EGF stimulation of HEK 293T cells transfected with Venus-RBDCRD quantified in Figure 2E. Scale bar is 20µm and applies to all panels. (TIFF) [file pone.0022607.s004.tif]

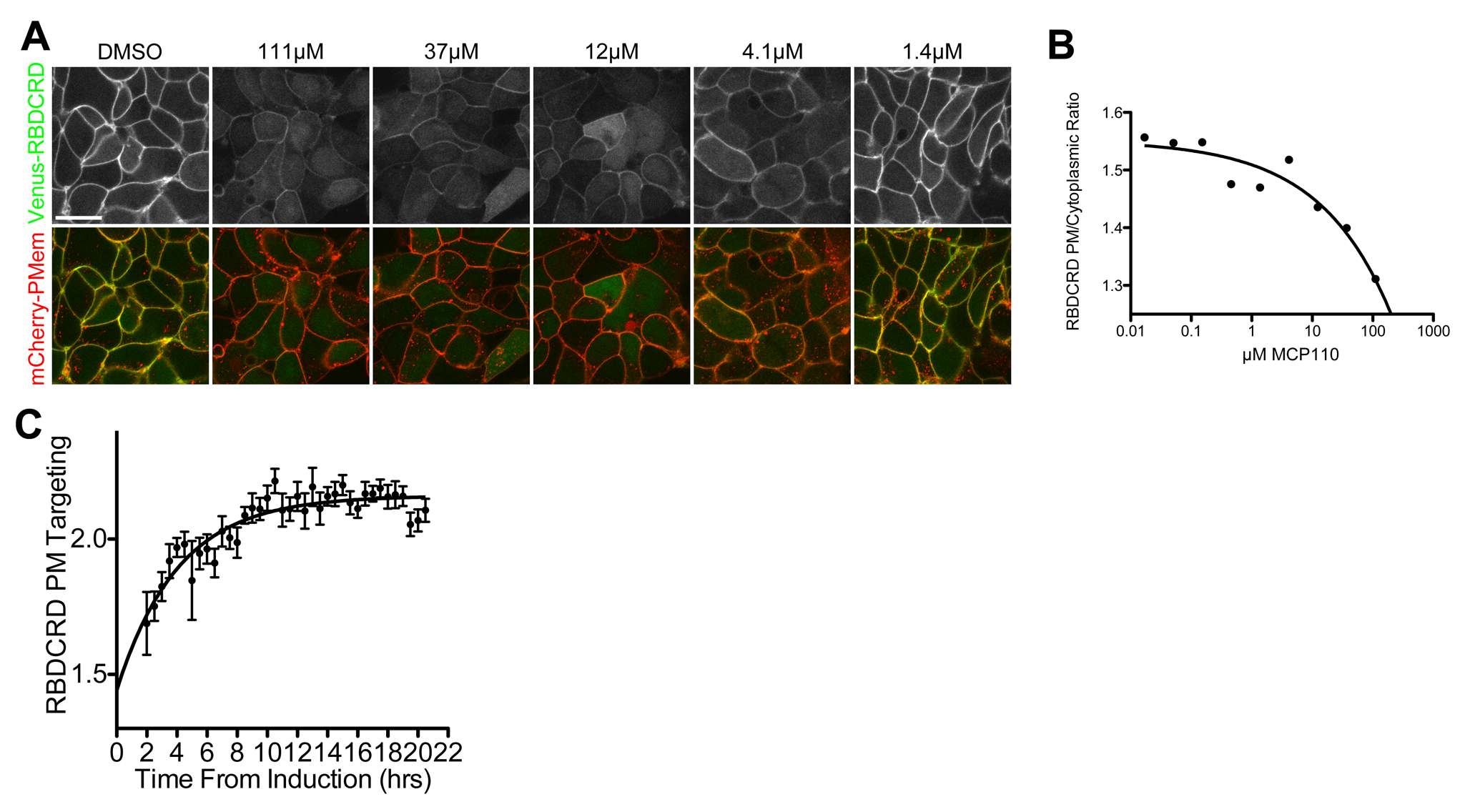

Supplement: Figure S5 — MCP110 disrupts RBDCRD PM targeting. (A) HEK 293 T-REx™ Venus-RBDCRD cell line was treated with MCP110 and images were acquired after 2.5hrs. Scale bar is 20µm and applies to all panels. (B) Venus-RBDCRD PM targeting was measured for cells dosed with MCP110. (C) HEK 293 T-REx™ Venus-RBDCRD cell line was induced with doxycycline and imaged every 30min, PM/cytoplasmic ratios of RBDCRD were then measured. (TIFF) [file pone.0022607.s005.tif]

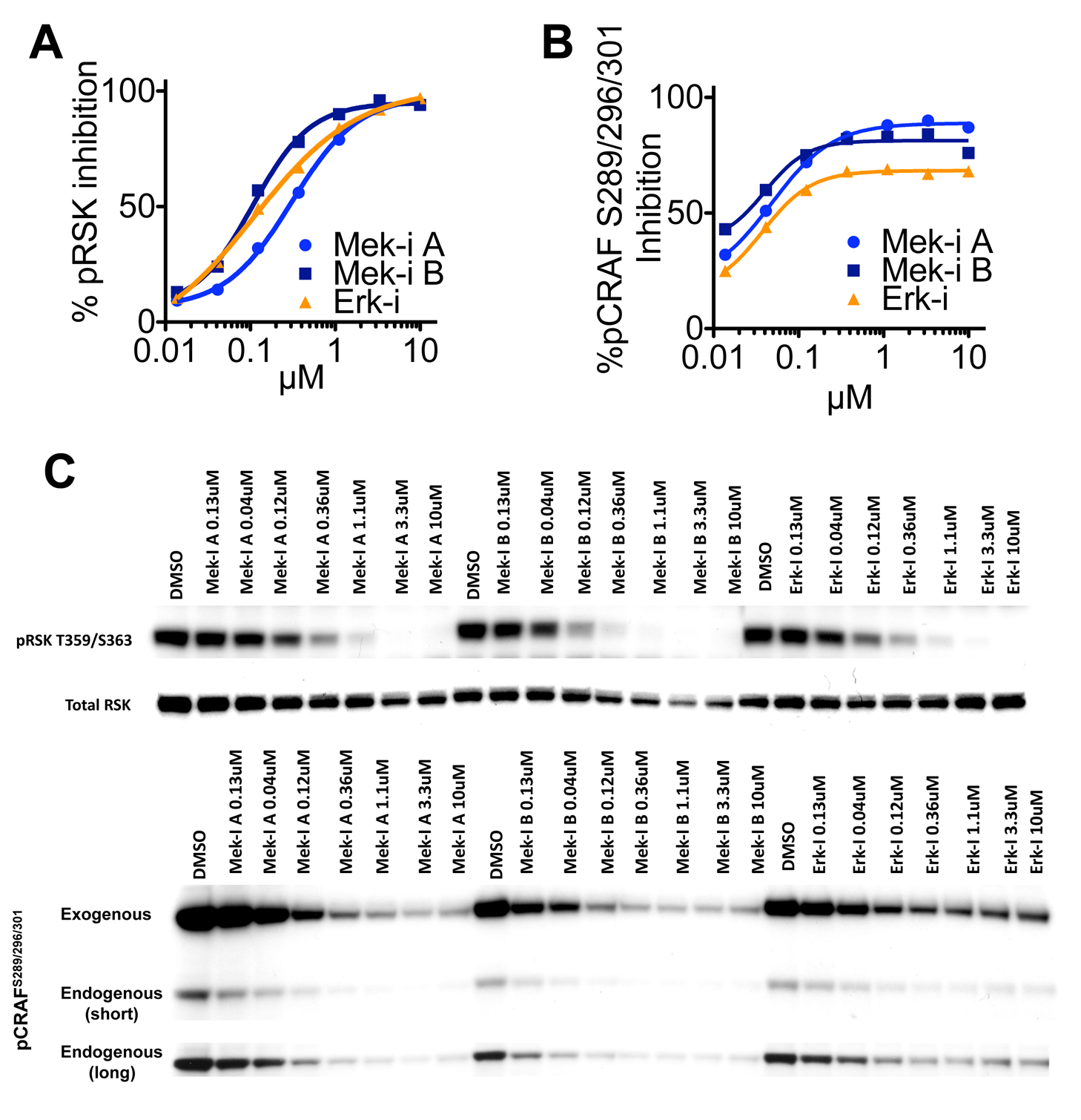

Supplement: Figure S6 — Dose dependent MAPK pathway inhibition by MEK (Mek-i A, Mek-I B) and Erk-i inhibitors (panel A) leads to release of negative feedback MAPK phosphorylation site on CRaf (panel B). Cells were treated with indicated inhibitor concentrations for 4 hours and lysates probed by immunoblot for pRSK (T359/363) and pCRaf(S289/296/301) levels (bottom). Curves represent quantitation of WB with Typhoon and curve fitting using Prism. (C,D) Western blot images of (A) and (B) respectively. (TIFF) [file pone.0022607.s006.tif]

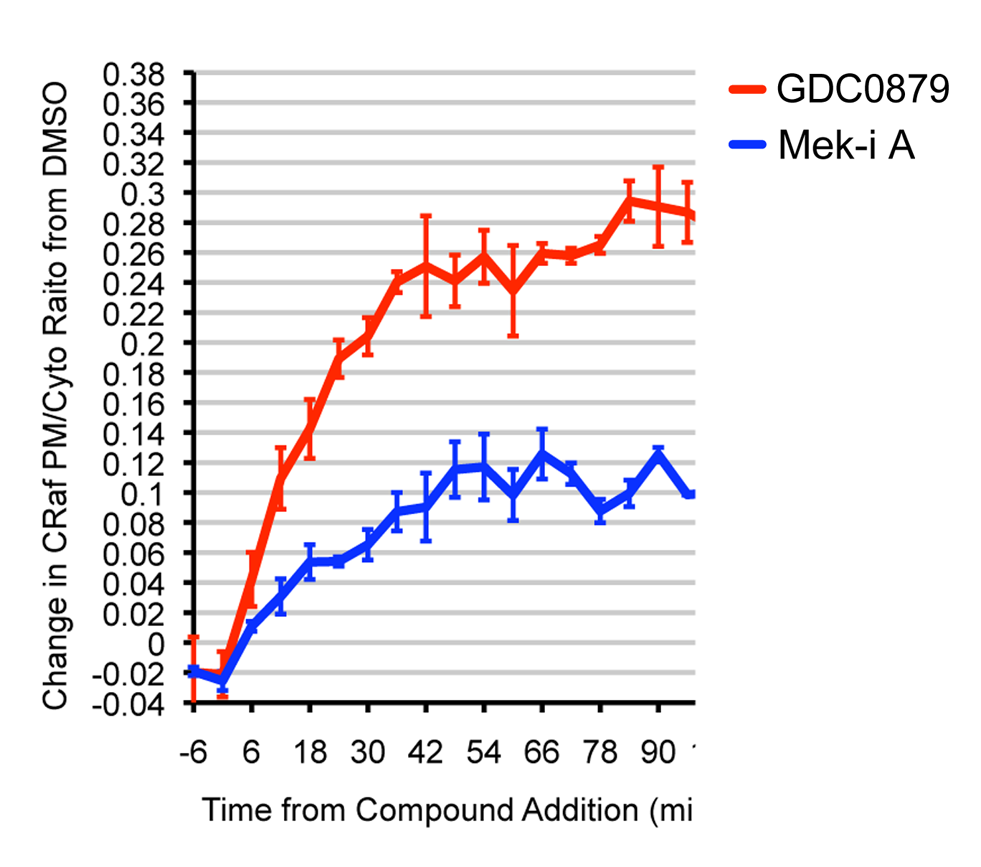

Supplement: Figure S7 — CRaf PM targeting is rapid with Mek-i A in KRasG12D/CRaf/Mek1/Erk2 cell line. MAPK cell line was imaged with time-lapse micrscopy and CRaf PM targeting was measured with the addition of 10µM GDC0879 or 10µM Mek-i A, images were acquired every 2min. (TIFF) [file pone.0022607.s007.tif]

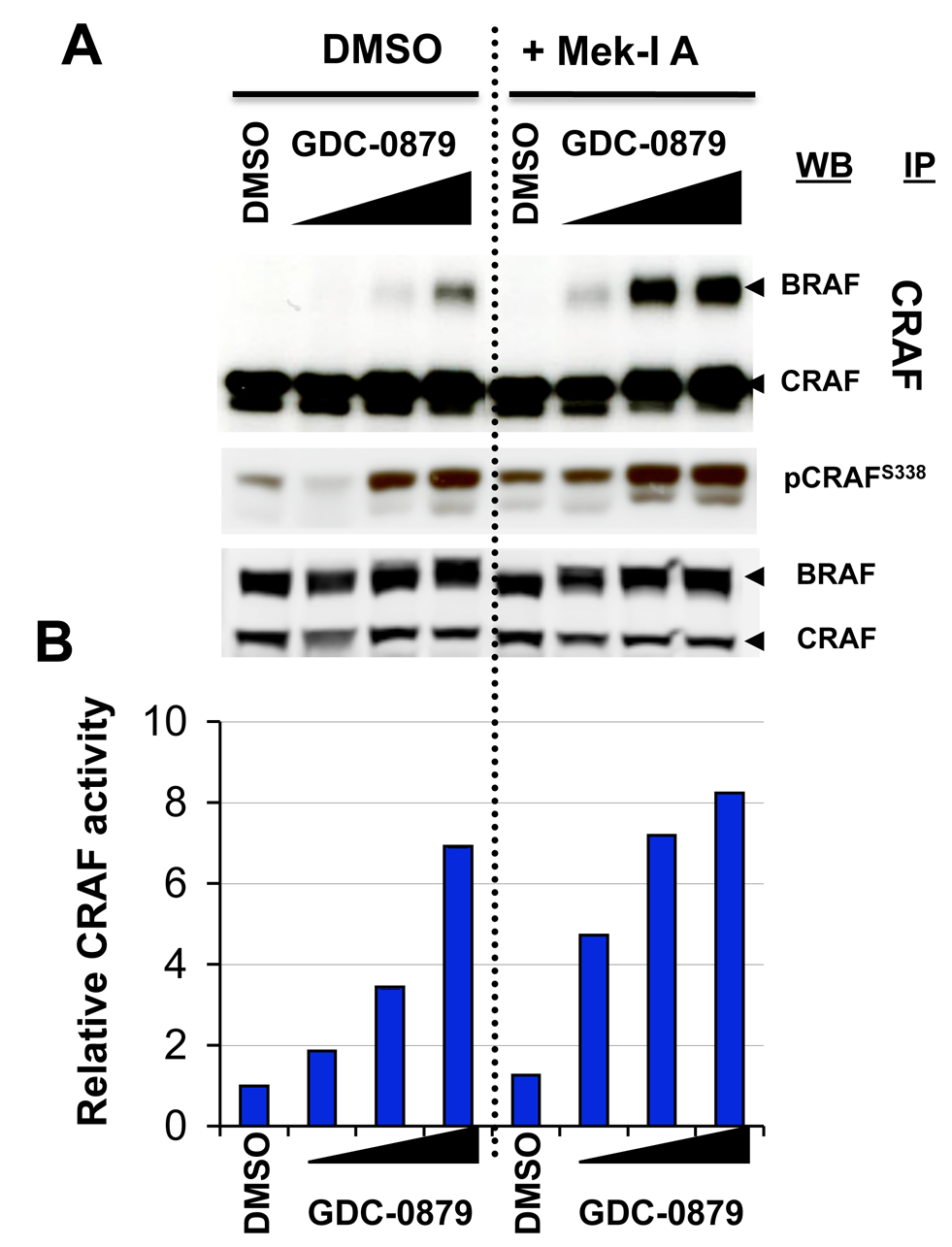

Supplement: Figure S8 — Additivity in targeting CRaf to the plasma membrane through combined priming by RAF inhibitors and negative feedback release by MEK inhibitors in H226 (KRasWT/CRafWT) NSCLC cells. Targeting CRaf to the plasma membrane upon combined priming by RAF inhibitors and negative feedback release by MEK inhibitors in H226 (KRasWT/CRafWT) NSCLC cells. (A) Top: Immunoblot of Braf:Craf heterodimers in H226 cells treated with GDC-0879 (0.1, 1, 10 µM) for 24 hours in cells pretreated for 1 hour with either DMSO or 1 µM Mek-i A. Heterodimer formation observed with Raf inhibitors alone is further increased upon co-administration of 1 μM MEK inhibitor Mek-i A. Bottom: Western blot of lysates from cells treated as above with indicated antibodies showing induction of pCRaf S338 and phospho-MEK levels. (B) CRaf IP kinase activity assays from lysates of cells treated as above. CRaf was immunoprecipitated from treated cells and kinase activity towards recombinant MEK was tested in vitro. Co-administration of Mek-i A with GDC-0879 resulted in a dose-dependent increase in maximal CRaf kinase activity across all Raf inhibitor doses. For the CRaf kinase activity assays, shown are relative phospho-MEK levels measured with MSD pMEK ELISA assay. (TIFF) [file pone.0022607.s008.tif]

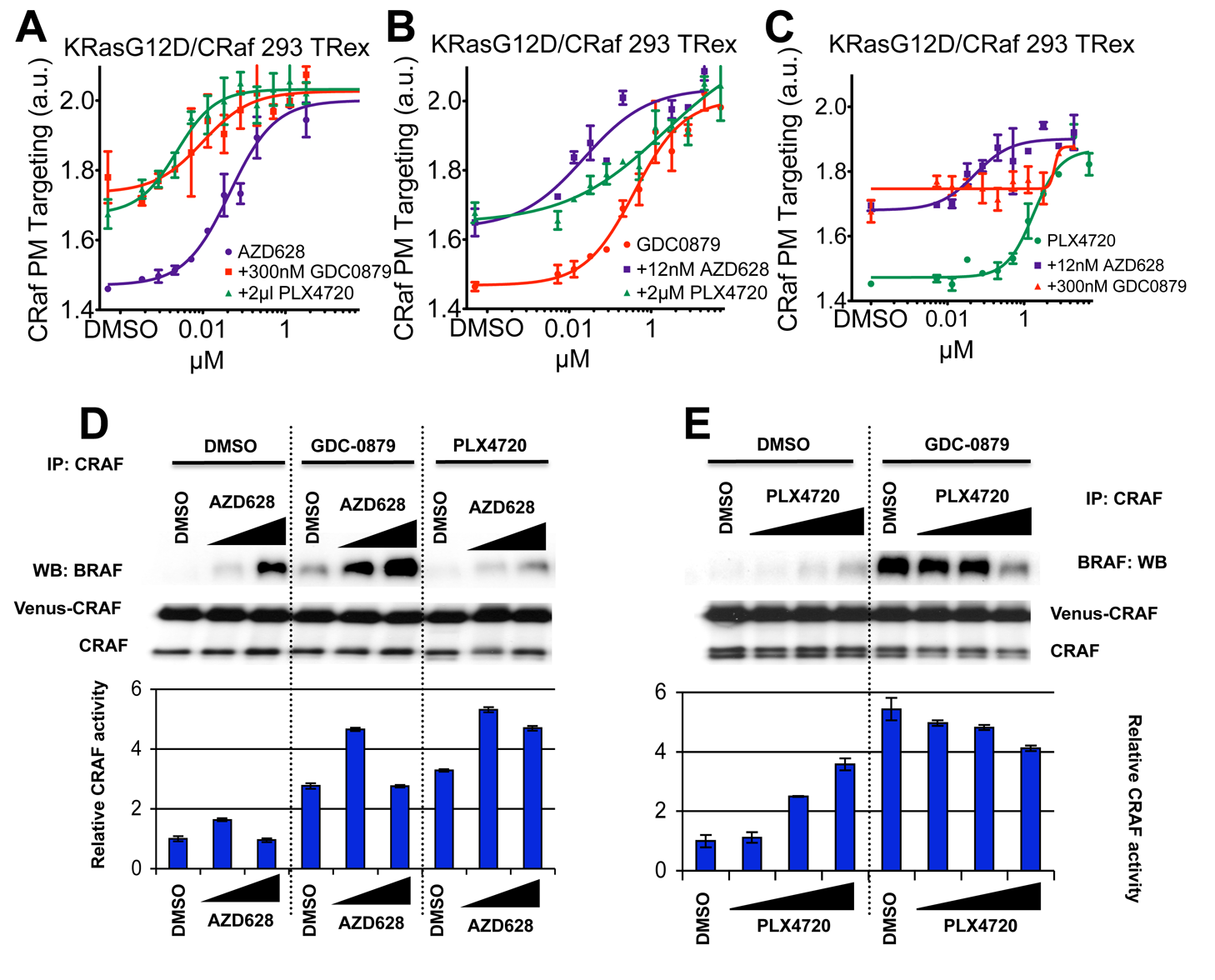

Supplement: Figure S9 — The DFG-out Raf inhibitor AZD-628 synergizes with the DFG-in inhibitors GDC-0879 and PLX4720 to potentiate CRaf activation in KRasG12D/CRaf 293 T-REx™ cells. Antagonism is observed between DFG-in Raf inhibitor GDC-0879 and DFG-in/c-helix shift PLX4720. (A) KRasG12D/CRaf HEK 293 T-REx™ cells were dosed with varying concentrations of AZD628 and constant dose of 2µM PLX4720 or 300nM GDC0879 and CRaf PM targeting was measured after 4hrs. (B) KRasG12D/CRaf HEK 293 T-REx™ cells were dosed with varying concentrations of GDC0879 and constant dose of 2µM PLX4720 or 12nM AZD628 and CRaf PM targeting was measured after 4hrs. (C) KRasG12D/CRaf HEK 293 T-REx™ cells were dosed with varying concentrations of PLX4720 and constant dose of 12nM AZD628 or 300nM GDC0879 and CRaf PM targeting was measured after 4hrs. (D) Top: Immunoblot of immunoprecipitated (IP) Craf shows Braf:Craf heterodimers in cells treated with AZD-628 (0.001 and 0.01) for 4 hours. Heterodimers are further induced in the presence of 50 nM GDC-0879 (middle panel) and reduced in the presence of 1 μM PLX4720, which disrupts heterodimer formation due to induction of a c-helix shift in BRaf and CRaf. Bottom: CRaf IP kinase activity assays from lysates of KRasG12D/Craf 293 T-REx™ cells treated with 0.001 and 0.01 µM AZD-628 for 4 hours. CRaf was immunoprecipitated from treated cells and kinase activity towards recombinant MEK was tested in vitro. (E) Top: Immunoblot of BRaf:CRaf heterodimers in cells treated with PLX4720 (0.1, 1, 10) for 4 hours. Weak heterodimer formation observed with PLX4720 alone, compared to treatment with 300 nM GDC-0879 alone (5th lane, DMSO/GDC-0879 panel). Increasing amounts of PLX4720 further destabilize BRaf:CRaf heterodimers due to distinct binding mode of PLX4720. Bottom: CRaf IP kinase activity assays from lysates of cells treated with PLX4720 (0.1, 1, 10) for 4 hours. CRaf was immunoprecipitated from treated cells and kinase activity towards recombinant MEK was tested in vitro. Adding PL [file pone.0022607.s009.tif]

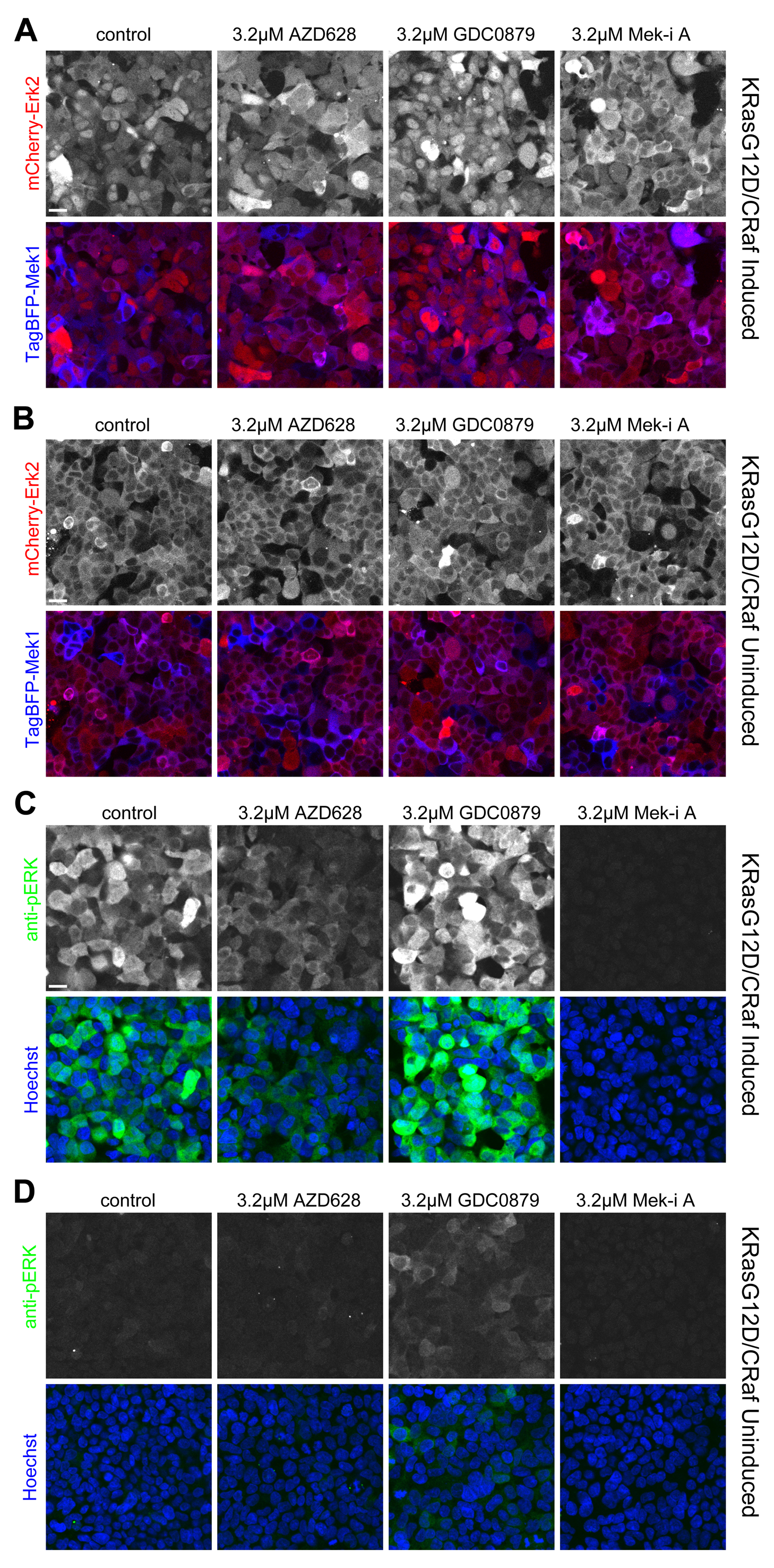

Supplement: Figure S10 — Images of mCherry-Erk1 localization and phospho-ERK. (A) Example images of mCherry-Erk2 sub-cellular localization, imaged 4hrs after inhibitor treatments with KRasG12D and CRaf induced. Scale bar is 20µm and applies to all panels. (B) Example images of mCherry-Erk2 sub-cellular localization, imaged 4hrs after inhibitor treatments with KRasG12D and CRaf uninduced. Scale bar is 20µm and applies to all panels. (C) Example images of pERK immuno-fluorescence, stained and imaged 5hrs after inhibitor treatments with KRas and CRaf induced. Scale bar is 20µm and applies to all panels. (D) Example images of pERK immuno-fluorescence, stained and imaged 5hrs after inhibitor treatments with KRas and CRaf uninduced. Scale bar is 20µm and applies to all panels. (TIFF) [file pone.0022607.s010.tif]
