# Supplementary material for: Live-Cell Microscopy Reveals Small Molecule Inhibitor Effects on MAPK Pathway Dynamics
Source: PLoS One. 2011 Aug 4;6(8):e22607. doi: 10.1371/journal.pone.0022607 (PMC3150364; doi:10.1371/journal.pone.0022607)
Supplement: Materials S1 — Matlab Programs. (DOCX) [file pone.0022607.s011.docx]

**Supplemental Material: Matlab Programs**

**Batch Script for analyzing PM targeting for a folder of images:**

Note: currently setup for parallel processing (toolbox required), replace parfor with for loop for linear processing

%%%% Copy write Daniel J Anderson 12/2010 Genentech %%%%

clc

clear

files = dir('*.tif');

parfor l = 1:numel(files)

I = imread(files(l).name);

handle = @adaptiveRadialScan;

[meanRatios nucCount] = handle(I);

PMRations(l,1) = meanRatios;

TotNuclei(l,1) = nucCount;

l

end

data(:,1) = PMRations(:);

data(:,2) = TotNuclei(:);

csvwrite('meanRatios.csv', data)

**Function to measure PM targeting for each image:**

%%%% Copy write Daniel J Anderson 12/2010 Genentech %%%%

function [meanRatios nucCount] = adaptiveRadialScan(I)

nucDiameter = 80;

nucIntensity = 0.07;

lineScans = 0;

lowerArea = 1500;

updderArea = 3000;

R = I(:,:,3);

G = I(:,:,2);

G = im2double(G);

%%Ras Membrane Masking

h = fspecial('gaussian',8,8);

Rttt = imfilter(R,h);

Rtot = zeros(size(R,1),size(R,1));

for thRtotsh = 1:49

Rt = im2bw(Rttt,thRtotsh/80);

RtotdDisk = strel('disk',5);

RtErode = imerode(Rt,RtotdDisk);

RtErode = imdilate(RtErode,RtotdDisk);

Rt = Rt-RtErode;

Rt = bwareaopen(Rt,10);

Rtot = Rtot+Rt;

Rtot(Rtot<0) = 0;

Rtot(Rtot>1) = 1;

end

Rtot = bwareaopen(Rtot,100);

Rtot = imdilate(Rtot,RtotdDisk);

RtotdDisk = strel('disk',6);

Rtot = imerode(Rtot,RtotdDisk);

Rtot = bwareaopen(Rtot,600);

PM = R;

neg = ~Rtot;

PM(neg) = 0;

%nuclear centroid finder

h = fspecial('gaussian',2,2);

Gnuc= imfilter(G,h);

BtTot = im2bw(Gnuc,1);

for NucThreshold = 1:60;

Bt = im2bw(Gnuc,NucThreshold/100);

Bt=~Bt;

Bt = bwareaopen(Bt,lowerArea);

BtBig = Bt;

BtBig = bwareaopen(BtBig,updderArea);

Bt = Bt-BtBig;

BtTot = BtTot + Bt;

end

L = bwlabel(BtTot);

s = regionprops(L, 'centroid');

centroids = cat(1, s.Centroid);

centroids = round(centroids(:,:));

nucCount = bwconncomp(BtTot);

nucCount = nucCount.NumObjects;

Rtot(Rtot<0)=0;

Rtot(Rtot>1)=1;

%Radial scanner

if centroids>0

nucCount = length(centroids(:,1));

else

lineScans = 0;

return

end

sizeX = length(R(1,:));

sizeY = length(R(:,1));

d=1;

stoRtotX=1;

stoRtotY=1;

hitCounter=1;

hitCountNorm=1;

totalScans=1;

hitCount=0;

xx=0;

for rA = 1:nucCount

jstart = centroids(rA,1);

istart = centroids(rA,2);

j = jstart;

i = istart;

degPM = 0;

countPM = 0;

rDeg = 0;

for rPM = 1:12

for rC = 1:nucDiameter

j = j+sind(degPM);

i = i-cosd(degPM);

if round(i)<1

break

end

if round(j)<1

break

end

if round(i)>(sizeY)

break

end

if round(j)>(sizeX)

break

end

if Rtot(round(i),round(j),1) == 1 && rC>(nucDiameter/3);

countPM = countPM+1;

break

break

end

%I(round(i),round(j),3) = 255;

rC = rC+1;

end

j = jstart;

i = istart;

degPM = degPM+30;

rPM = rPM+1;

if countPM == 6

for rD = 1:180

iA = i;

iAA = i;

iB = i;

iC = i;

iD=i;

iE=i;

jA = j;

jAA = j;

jB = j;

jC = j;

jD=j;

jE=j;

for rC = 1:round(nucDiameter)

j = j+sind(rDeg);

i = i-cosd(rDeg);

if round(i)<1

break

end

if j<1

break

end

if round(i)>(sizeY)

break

end

if round(j)>(sizeX)

break

end

jE=jD;

jD=jC;

jC=jB;

jB=jA;

jA=jAA;

jAA=j;

iE=iD;

iD=iC;

iC=iB;

iB=iA;

iA=iAA;

iAA=i;

if Rtot(round(iB),round(jB)) == 1 && rC>(nucDiameter/2) && G(round(iE),round(jE))<0.95 && G(round(iE),round(jE))>0.001 && G(round(iAA),round(jAA))>0.001

cytoRatio=((G(round(iAA),round(jAA)))/((G(round(iE),round(jE)))));

lineScans(stoRtotY)=cytoRatio;

stoRtotY=stoRtotY+1;

totalScans = totalScans+1;

break

end

rC = rC+1;

end

j = jstart;

i = istart;

rDeg = rDeg+2;

rD = rD+1;

end

break

end

end

end

meanRatios = mean(lineScans);

**Batch script for measuring nuclear/cytoplasmic Erk distribution**

clc

clear

files = dir('*.tif');

%for l = 1:4

for l = 1:numel(files)

I = imread(files(l).name);

handle = @nuclearErk;

[meanRatio] = handle(I);

totErk(l) = meanRatio;

meanRatio

end

csvwrite('meanRatios.csv', totErk)

**Sub-batch script for measuring nuclear/cytoplasmic Erk distribution**

Note: currently setup for parallel processing (toolbox required), replace parfor with for loop for linear processing

clc

clear

files = dir('*.tif');

%for l = 1:4

for l = 1:numel(files)

I = imread(files(l).name);

handle = @nuclearErk;

[meanRatio] = handle(I);

totErk(l) = meanRatio;

meanRatio

end

csvwrite('meanRatios.csv', totErk)

function [meanRatio] = nuclearErk(I)

lowerArea = 200;

updderArea = 700;

blurPixels = 2;

B = I(:,:,3);

B = im2double(B);

h = fspecial('gaussian',blurPixels,blurPixels);

B= imfilter(B,h);

BtTot = im2bw(B,1);

R = I(:,:,2);

G = I(:,:,2);

Gnuc = B;

h = fspecial('gaussian',3,3);

Gnuc= imfilter(Gnuc,h);

BtTot = im2bw(Gnuc,1);

for NucThreshold = 2:50;

Bt = im2bw(Gnuc,NucThreshold/200);

Bt=~Bt;

Bt = bwareaopen(Bt,lowerArea);

BtBig = Bt;

BtBig = bwareaopen(BtBig,updderArea);

Bt = Bt-BtBig;

L = bwlabel(Bt);

s = regionprops(L,'Perimeter', 'Area');

Perm_values = [s.Perimeter];

Perm_values = double(Perm_values);

Area_values = [s.Area];

Area_values = double(Area_values);

Area_values = sqrt(Area_values);

Ratio_values = Perm_values./Area_values;

idx = find(6 > Ratio_values);

Bt = ismember(L, idx);

BtTot = BtTot + Bt;

end

BtTot = bwareaopen(~BtTot,150);

BtTot = ~BtTot;

nucCount = bwconncomp(BtTot);

nucCount = nucCount.NumObjects;

cc = bwconncomp(BtTot);

totRatio = 0;

parfor A = 1:nucCount

[ErkRatio] = nuclearErksub(BtTot,R,A,I)

totRatio(A) = ErkRatio;

end

meanRatio = mean(totRatio);

**Function to measure nuclear/cytoplasmic Erk distribution**

function [ErkRatio] = nuclearErksub(BtTot,R,A,I)

cc = bwconncomp(BtTot);

subI = false(size(BtTot));

subI(cc.PixelIdxList{A}) = true;

CC = bwconncomp(subI);

L = regionprops(CC,R,'MeanIntensity');

intensity = cat(1,L.MeanIntensity);

nuclearmCherry = mean(intensity);

GeDisk = strel('disk',3);

Ring = imdilate(subI,GeDisk);

Ring = Ring-subI;

CC = bwconncomp(Ring);

L = regionprops(CC,R,'MeanIntensity');

intensity = cat(1,L.MeanIntensity);

cytomCherry = mean(intensity);

ErkRatio = nuclearmCherry/cytomCherry;
